# Neurological adverse events of PD-1/PD-L1 immune checkpoint inhibitors in clinical trials: A meta-analysis

**DOI:** 10.1016/j.clinsp.2025.100698

**Published:** 2025-07-10

**Authors:** Yanting Zhou, Hansong Yu, Hongyan Li

**Affiliations:** General Surgery Department, Xuanwu Hospital, Beijing, China

**Keywords:** Immunotherapy, Neurotoxicity syndromes, Immune-related adverse events, Immune checkpoint inhibitors, Cancer neuroscience

## Abstract

**Objective:**

Based on the data in clinical studies, the authors explored the potential link between cancer immunotherapy and Neurological Adverse Events (NAEs), and established a clinical picture.

**Methods:**

The authors searched PubMed, Embase, Cochrane Library, Web of Science databases, and ClinicalTrials.gov for Randomized Controlled Trials (RCTs) using PD-1/PD-L1 inhibitors for cancer until November 2023. A total of 141 articles were included, covering 12 PD-1/PD-L1 inhibitors and 18 cancer types.

**Results:**

Finally, 90,079 patients of 141 RCTs met the eligibility criteria. Data showed no significant difference in the incidence of NAEs in patients receiving PD-1/PD-L1 inhibitors compared to the control group (OR = 1.07; 95 % CI 0.95, 1.21; *p* = 0.25). However, the authors surprisingly found that the incidence of serious NAEs in the PD-1/PD-L1 group was higher than the control group (OR = 1.34; 95 % CI 1.24, 1.44; *p* < 0.00001), the same with NAEs in atezolizumab subgroup (OR = 1.66; 95 % CI 1.08, 2.56; *p* = 0.02). It is worth noting that the intra-group heterogeneity of the serious NAEs subgroup was relatively small (*I*^2^ = 16 %, *p* = 0.06).

**Conclusion:**

The authors first proposed the opinion that the incidence of serious NAEs in immunotherapy patients was significantly higher than in other groups, providing a novel direction for research.

## Highlights

• A meta-analysis of 141 RCTs evaluated 12 PD-1/PD-L1 inhibitors in 18 cancer types.
• PD-1/PD-L1 therapy has a higher incidence of serious Neurological Adverse Events (NAEs) compared to the control group.
• Six predominant NAEs showed higher incidence in the PD-1/PD-L1 group, including stroke and Guillain-Barré.
• This analysis reveals a potential interaction between cancer immunotherapy and the nervous system.

## Introduction

Cancer has always been a challenging problem that scientists have been committed to tackling. The advent of immune checkpoint inhibitors in recent years has undoubtedly brought us new perspectives and hope. Unlike traditional chemotherapy, immunotherapy targets the immune escape of cancer cells. It blocks the binding of immune checkpoints and ligands through drugs, thereby relieving collective immune suppression and reactivating autoimmune cells to exert anti-tumor effects.^1^ This type of drug has greatly changed the present situation of cancer treatment, showing lasting clinical responses in a variety of cancers and, therefore recommended as a first-line treatment for a variety of cancers.^2^^,^^3^

PD-1/PD-L1 inhibitors are among the most commonly used immune checkpoint inhibitors in clinical practice. As the scope of use expands, its unique adverse reaction spectrum has also received attention.^4^^,^^5^ Among them, although the incidence of NAEs is not high, it is difficult to diagnose and brings a poor prognosis. Related case reports and review articles have been published and received attention in recent years.^6^, ^7^, ^8^ However, existing studies are still mostly limited to individual cases or a certain type of NAEs. Research that explores NAEs of PD-1/PD-L1 inhibitors in the real world is still inadequate. However, numerous clinical trials involving various PD-1/PD-L1 inhibitor drugs have yielded valuable data in recent years, which contains information on NAEs, providing substantial information for future research in this area.

Existing studies have explored the NAEs of immune checkpoint inhibitors through review and meta-analysis.^9^, ^10^, ^11^ Still, most of them are qualitative descriptions based on specific types of NEAs in related but limited publications. These studies are undoubtedly very valuable, but due to different research methods, different drugs, and different types of cancer, they are prone to outdated and contradictory conclusions. Against this background, the authors aimed to review and analyze the literature systematically, conducting the latest and most comprehensive analysis of NAEs in randomized controlled trials. Through these efforts, the authors strive to establish a clinically relevant picture of PD-1/PD-L1 inhibitors’ neurotoxicity, for exploring the potential connection between the nervous system and cancer immunotherapy.

## Methods

This study was designed and reported following the PRISMA^12^ standard (eTable 1) and the Assessing the Methodological Quality of Systematic Reviews^13^ (AMSTAR) (eFig. 1). In addition, the authors have registered this study in PROSPERO (registration number CRD42023479508). After completing the work above, two researchers (YZ, and HY) independently performed a literature search, eligibility assessment, data extraction, and qualitative assessment. Any inconsistencies in the search results were resolved through group discussion until a consensus was reached, and any remaining doubts were reported to HL, who reviewed and made the final decision.

### Data sources and search strategy

This study conducted a comprehensive search for RCTs using PD-1/PD-L1 inhibitors to treat different types of cancers in databases including PubMed, Embase, Cochrane Library, and Web of Science from the inception of each database to November 27, 2023. In addition, the authors reviewed Clinical Trails.gov to determine the latest data for each clinical trial if the ClinicalTrials.gov ID was contained in the literature. Two researchers (YZ and HY) searched the databases independently. The Pubmed screening formula is as follows, and the remaining database search formulas are listed in the supplementary content (eMethods): (("cancer"[All Fields] OR "tumor"[All Fields] OR " tumor "[All Fields] OR "neoplasm"[All Fields] OR "carcinoma"[All Fields]) AND ("immune checkpoint inhibitor"[All Fields] OR "programmed death receptor 1″[All Fields] OR "programmed cell death 1″[All Fields] OR "anti-PD-1″[All Fields] OR "PD-1″[All Fields] OR "anti -PD-L1"[All Fields] OR "PD-L1"[All Fields] OR "nivolumab"[All Fields] OR "pembrolizumab"[All Fields] OR "atezolizumab"[All Fields] OR "durvalumab"[All Fields] OR "avelumab"[All Fields] OR "immunotherapy"[All Fields]) AND ("irAEs "[All Fields] OR "immune-related adverse events"[All Fields] OR "treatment-related adverse events"[All Fields] OR "treatment-related AEs"[All Fields] OR "select adverse events"[All Fields] OR "select AEs"[All Fields] OR" immune-mediated adverse events"[All Fields] OR "immune-mediated AEs"[All Fields])) AND ((clinicaltrialphaseii [Filter] OR clinicaltrialphaseiii [Filter] OR clinicaltrialphaseiv [Filter] OR controlledclinicaltrial [Filter] OR randomizedcontrolledtrial [Filter]) AND (humans[Filter])).

**Fig. 1:**
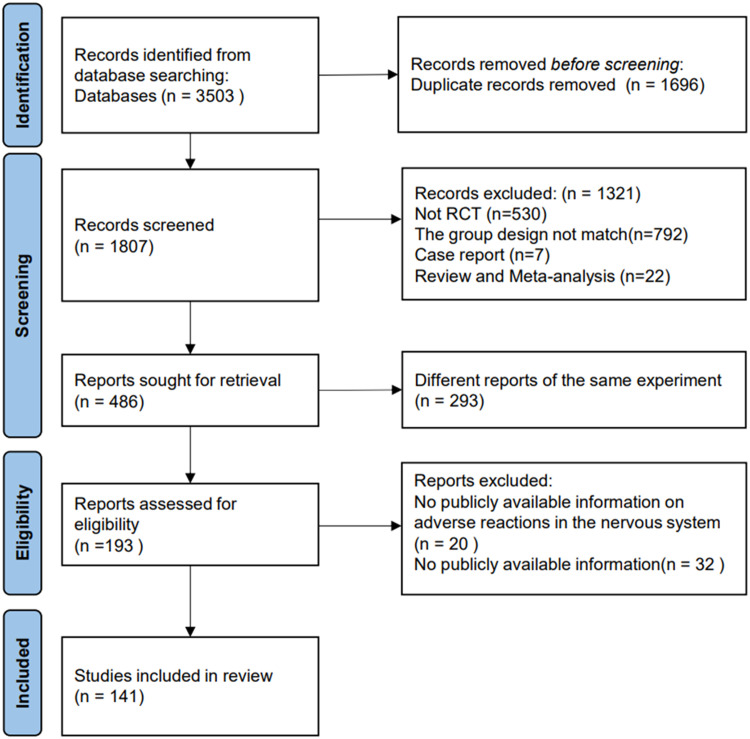
PRISMA flow diagram of study selection for this meta-analysis.

In addition, the authors expanded the search and reviewed the references included in the retrieved articles. The authors merged the search results into bibliographic management tools (EndNote and Zotero) and eliminated duplicates using the Bramer method. The literature search and reference list review identified 1807 relevant publications (Fig. 1).

### Eligibility criteria and study selection

Included studies were RCTs using PD-1/PD-L1 inhibitors to treat patients with solid tumors. Combined and monotherapy were both included, and the control group did not contain PD-1/PD-L1 inhibitors, such as chemotherapy drugs, placebo, etc. The primary endpoint was to evaluate the difference in the incidence of neurological toxicity reported between cancer patients who received and did not receive PD-1/PD-L1 inhibitors. The study aims to respond to the question: Is the incidence of NAEs different for PD-1/PD-L1-based immunotherapy? The acronym PICO was used, which corresponds to the areas P (population), I (intervention), C (comparison) and O (outcome):

1. Population: Patients with solid tumors;
2. Intervention: PD-1/PD-L1 inhibitors, combined and monotherapy were both included;
3. Comparison: PD-1/PD-L1 inhibitors not included, such as chemotherapy drugs, placebo;
4. Outcome: The difference in the incidence of neurological toxicity reported

The authors reviewed each publication and its final results registered on clinicaltrials.gov(clinicaltrials.gov), and only the latest and most complete clinical trial reports for the same clinical trial were included. Specific inclusion criteria are listed in Table 1. Finally, 90,079 patients in 141 RCTs (published between 2016 and 2023) met the eligibility criteria and were included.^14^, ^15^, ^16^, ^17^, ^18^, ^19^, ^20^, ^21^, ^22^, ^23^, ^24^, ^25^, ^26^, ^27^, ^28^, ^29^, ^30^, ^31^, ^32^, ^33^, ^34^, ^35^, ^36^, ^37^, ^38^, ^39^, ^40^, ^41^, ^42^, ^43^, ^44^, ^45^, ^46^, ^47^, ^48^, ^49^, ^50^, ^51^, ^52^, ^53^, ^54^, ^55^, ^56^, ^57^, ^58^, ^59^, ^60^, ^61^, ^62^, ^63^, ^64^, ^65^, ^66^, ^67^, ^68^, ^69^, ^70^, ^71^, ^72^, ^73^, ^74^, ^75^, ^76^, ^77^, ^78^, ^79^, ^80^, ^81^, ^82^, ^83^, ^84^, ^85^, ^86^, ^87^, ^88^, ^89^, ^90^, ^91^, ^92^, ^93^, ^94^, ^95^, ^96^, ^97^, ^98^, ^99^, ^100^, ^101^, ^102^, ^103^, ^104^, ^105^, ^106^, ^107^, ^108^, ^109^, ^110^, ^111^, ^112^, ^113^, ^114^, ^115^, ^116^, ^117^, ^118^, ^119^, ^120^, ^121^, ^122^, ^123^, ^124^, ^125^, ^126^, ^127^, ^128^, ^129^, ^130^, ^131^, ^132^, ^133^, ^134^, ^135^, ^136^, ^137^, ^138^, ^139^, ^140^, ^141^, ^142^, ^143^, ^144^, ^145^, ^146^, ^147^, ^148^, ^149^, ^150^, ^151^, ^152^, ^153^, ^154^

**Table 1:** Literature inclusion criteria.

Category	Criteria
Language	English
Patient	Adults (≥ 18) with solid tumors
Study type	Randomized Controlled Trial (RCT)
Experimental Group	Including PD-1/PD-L1 inhibitor
Control Group	Without PD-1/PD-L1 inhibitor
Outcome	Neurological Adverse Events Reported

### Data extraction

Two researchers (YZ and HY) independently conducted data extraction, which included gathering basic information and NAEs data from ClinicalTrials.gov and Supplementary Materials of the 141 selected RCT publications. Discrepancies were reviewed by another investigator on the team (HL) and resolved by consensus. The general information on publications and the baseline characteristics of the patients were summarized in Supplementary (eTable 2). Since most adverse events data came from Clinical Trails.gov, the NAEs were classified into serious and other adverse reactions based on the standards of the website. In addition, the authors also merged synonyms for adverse reactions (such as Dysgeusia and Ageusia).

### Endpoint setting and stratification strategy

**Fig. 2:**
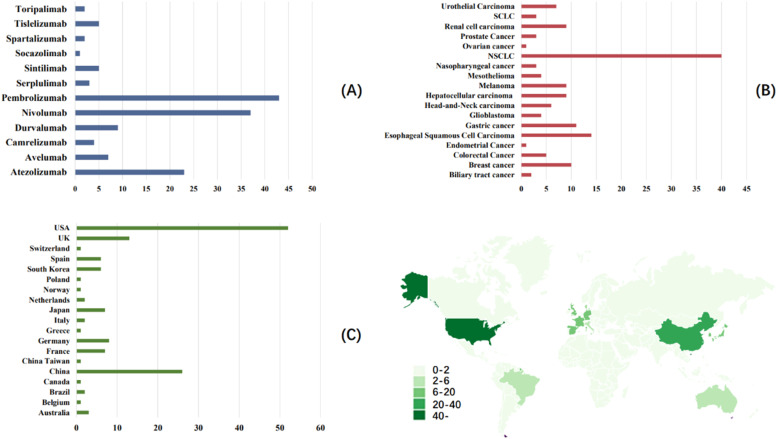
Summary of neurological subgroup analysis of included literature. Type of PD-1/PD-L1 drug used (A), cancer type (B), country of clinical study publications (C).

The primary outcome was to compare whether the incidence of neurological toxicity after PD-1/PD-L1 immunotherapy was different from that of other chemotherapy-based treatments. The data of varying intervention groups in the same study were extracted and reported separately. In order to more comprehensively analyze the impact of various factors on the adverse reactions of the nervous system after PD-1/PD-L1 immunotherapy, the authors performed subgroup analysis based on countries, cancer types, PD-1/PD-L1 inhibitor types, and severity of adverse reactions of the nervous system. Fig. 2 details the stratification strategy the authors adopted for subgroup analysis.

### Data synthesis and analysis

The authors calculated the overall event rate by dividing the number of patients with all neurotoxicity in the included trials by the total number of patients. Moreover, the authors counted and summarized the number of events for each type of neurological toxicity of interest to provide a more comprehensive understanding of each type of NAEs. Due to the inherent heterogeneity among the included trials, the authors used a Random-Effects Model (RE) to estimate the Odds Ratio (OR) and its corresponding 95 % Confidence Interval (95 % CI). The *I*^2^ index and Cochran Q statistic were used to examine the heterogeneity between trials for each outcome.^155^ When significant heterogeneity (*I*^2^ > 50 %) is detected, the authors will perform a subgroup analysis and discuss its reasons.^156^ For subgroups with less heterogeneity (*I*^2^ < 50 %), the authors will use a fixed-effects model and conduct further evaluation and comparison. Publication bias was assessed by funnel plots and risk of bias was summarized in Supplement (eTable 3). All reported p-values were two-sided, and *p* < 0.05 was considered statistically significant. Additionally, the authors employed the GRADE assessment[156] to evaluate the evidence pertaining to the interventions. Subgroup analyses were performed according to tumor type, country, and PD-1/PD-L1 inhibitor. Data analysis and visualization were performed using Microsoft Office Excel, Review Manager 5.4, Origin2021, GraphPad Prism 9.5, and other software.

## Incidence rate of neurological adverse events in the PD-1/PD-L1 inhibitors group

**Fig. 3:**
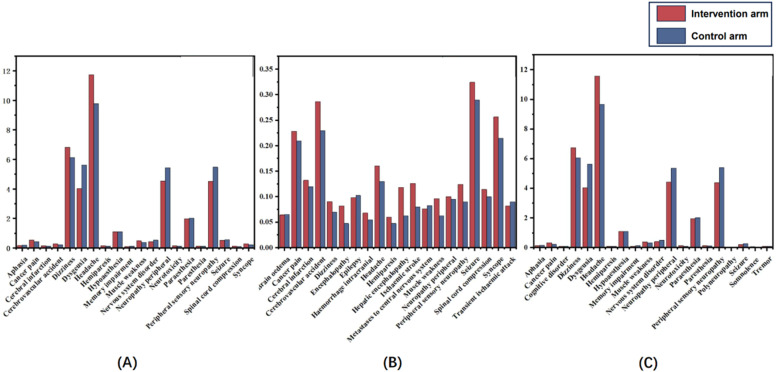
Statistical chart of the incidence of neurological adverse events in the included studies. All neurological adverse events (A), serious neurological adverse events (B), non-serious neurological adverse events (C).

The incidence of NAEs is defined as the number of patients exposed to neurological toxicity divided by the total number of patients in the corresponding intervention or control group. In the included studies, a total of 49,979 cancer patients were exposed to PD-1/PD-L1 inhibitors. Among them, at least 20,436 (40.89 %) experienced neurological adverse events of varying degrees, of which 2049 (4.10 %) had 1 serious neurological adverse reaction. Statistics show that the most common NAE is Headache (11.72 %), followed by Dizziness (6.83 %) and Peripheral sensory neuropathy (4.51 %). The incidence of Seizure (0.63 %) ranked first among the patients' serious neurological adverse reactions. Still, the authors believe this may be highly correlated with the two included RCTs of glioblastoma, followed by Cerebrovascular accident (0.28 %) and Syncope (0.25 %). Fig. 3 shows the incidence of different types of neurological adverse effects in the intervention and the control groups.

## Neurological adverse events spectrum of PD-1/PD-L1 inhibitors

### Overall of toxicities

Among the 141 included clinical studies, the authors first compared and calculated the overall incidence of neurotoxicity. The authors found that the heterogeneity between studies was high (*I*^2^ = 91 %, *p* < 0.00001), so a random effects model was used. It was found that the incidence of NAEs in cancer patients treated with PD-1/PD-L1 inhibitors was not statistically different from that in patients who did not use (*p* = 0.25). Based on the GRADE guidelines, the level of evidence for this conclusion is moderate. Considering that the high heterogeneity reduced the compatibility between studies, the authors next conducted subgroup analysis as comprehensively as possible. The authors visualized the basic situation of the subgroups so that readers could understand it intuitively (Fig. 2). Forest plots of all studies and subgroups are shown in the Supplementary Materials (eFig. 2).

### Subgroup by severity of toxicities

Initially, the authors performed a subgroup analysis of NAEs according to severity. Interestingly, the authors found that when the investigation content became the incidence of serious neurological adverse events, the heterogeneity between studies became smaller (*I*^2^ = 16 %, *p* = 0.06), so the fixed-effect model could be used for analysis. Based on this, the authors speculated that the severity of adverse reactions was one of the important factors affecting consistency. It is worth noting that the incidence of serious NAEs in patients using immunotherapy targeting PD-1/PD-L1 was 1.34 times that of patients not using it (*p* < 0.00001), which runs counter to the results of previous studies that the overall adverse reactions of PD-1/PD-L1 inhibitors were significantly lower than those of the control group.^157^^,^^158^ To explore the potential connection between serious neurological adverse reactions and immunotherapy, the authors further analyzed serious NAEs in specific types.

**Fig. 4:**
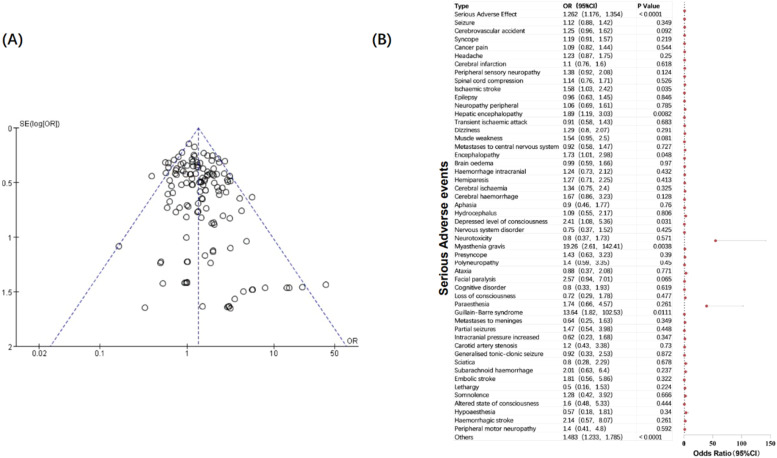
Funnel plot (A) and forest plot (B) and of serious NAEs.

The authors combined all serious NAEs in the included studies, calculated the incidence of each type of neurological toxicity, and compared the differences between the two groups (Fig. 4). The authors found that patients treated with PD-1/PD-L1 inhibitors had a higher risk of serious neurological toxicity compared with patients in the control group. Specifically, the incidence of serious neurological adverse reactions such as ischaemic stroke (OR = 1.58; 95 % CI 1.03, 2.42), hepatic encephalopathy (OR = 1.89; 95 % CI 1.19, 3.03), encephalopathy (OR = 1.73; 95 % CI 1.01, 2.98), depressed level of consciousness (OR = 2.41; 95 % CI 1.08, 5.36), myasthenia gravis (OR = 19.26; 95 % CI 2.61, 142.41), and Guillain-Barre syndrome (OR = 13.64; 95 % CI 1.82, 102.53) in PD-1/PD-L1 immunotherapy was significantly higher than that in the control group (*p* < 0.05). It is worth noting that the incidence of Myasthenia gravis and Guillain-Barre syndrome in the immunotherapy group was extremely higher than that in the control group. These two adverse reactions are also often reported in clinical case reports. The authors speculate that they may be related to the etiology of the two diseases, for mistakenly activates autoimmunity. They are immune-related adverse reactions and are relatively rare in other cancer treatments. The reasons for the increased incidence of other nervous system adverse reactions, mainly diseases of the central nervous system, need further exploration.

### Subgroup by PD-1/PD-L1 inhibitor types

The study included 12 different types of PD-1/PD-L1 inhibitor types, namely Pembrolizumab (*n* = 43), Nivolumab (*n* = 37), Atezolizumab (*n* = 23), Durvalumab (*n* = 9), Avelumab (*n* = 7), Sintilimab (*n* = 5), Tislelizumab (*n* = 5), Camrelizumab (*n* = 4), Serplulimab (*n* = 3), Spartalizumab (*n* = 2), Toripalimab (*n* = 2) and Socazolimab (*n* = 1). Subgroup analysis was performed on studies with *n* ≥ 10, and the heterogeneity within the three subgroups was large, for which the random effects model was used. Among them, the heterogeneity of the atezolizumab subgroup was high, but the incidence of NAEs in the immunotherapy group was higher than that in the control group (*p* = 0.02). The authors speculate that its heterogeneity may mainly come from the differences in cancer types and the grading of adverse reactions.

### Subgroup by cancer type

The 141 studies included Non-Small Cell Lung Cancer (NSCLC) (*n* = 40), Esophageal Squamous cell carcinoma (*n* = 14), Gastric cancer (*n* = 11), Breast cancer (*n* = 10), Hepatocellular carcinoma (*n* = 9), Melanoma (*n* = 9), Renal cell carcinoma (*n* = 9), Urothelial carcinoma (*n* = 7), Head-and-Neck carcinoma (*n* = 6), Colorectal cancer (*n* = 5), Glioblastoma (*n* = 4), Mesothelioma (*n* = 4), Nasopharyngeal cancer (*n* = 3), Prostate cancer (*n* = 3), SCLC (*n* = 3), Biliary tract cancer (*n* = 2), Endometrial cancer (*n* = 1), Ovarian cancer (*n* = 1), a total of 18 cancer types (Fig. 2B). The authors performed subgroup analysis on cancers with *n* ≥ 10 included studies, and forest plots showed that the heterogeneity of each cancer subgroup was large. The random effects model showed no difference in the incidence of neurological adverse reactions among different cancers (*p* > 0.05).

### Subgroup by country

Subgroup data were collected based on the country information of the first author of the included literature. A total of 19 locations were included in the 141 studies (Fig. 2C). They were USA (*n* = 52), China (*n* = 26), UK (*n* = 13), Germany (*n* = 8), France (*n* = 7), Japan (*n* = 7), South Korea (*n* = 6), Spain (*n* = 6), Australia (*n* = 3), Brazil (*n* = 2), Italy (*n* = 2), Netherlands (*n* = 2), Belgium (*n* = 1), Canada (*n* = 1), China Taiwan (*n* = 1), Greece (*n* = 1), Norway (*n* = 1), Poland (*n* = 1), Switzerland (*n* = 1). Subgroup analysis of countries with *n* ≥ 10 showed that in the subgroups of the first three regions, the incidence of neurological adverse reactions in the PD-1/PD-L1 inhibitor treatment group was not significantly different from that in the control group, and the heterogeneity was large (*p* > 0.05).

## Strengths and limitations

Compared to the clinical efficacy of drugs, adverse reactions have always been a relatively neglected part. However, the occurrence of NAEs always results in the termination of treatment regimens and leads to undesirable prognosis effects.^159^ Clinical research on NAEs of the drugs targeting PD-1/PD-L1 is undoubtedly lacking, and existing studies published were basically in the form of case reports or reviews.^160^^,^^161^ The quality of evidence from previous studies was relatively low due to the few included studies. With the large-scale implementation of phase III clinical trials of immunotherapy in recent years, a rich clinical data resource has been provided for the study of NAEs, and it is meaningful to include more clinical research results for analysis. This study has well supplemented the gap in this area. Not only did it comprehensively incorporate relevant data from so many existing clinical studies, it also proposed the conclusion for the first time that the incidence of serious NAEs in immunotherapy was higher than that in the control group. Furthermore, the authors summarized the incidence of specific adverse reaction types, which provided ideas and data for the subsequent exploration of the relationship between neurological adverse reactions and PD-1/PD-L1 immunotherapy, further between the nervous system and cancer treatment.

Excitingly, this research also complements the discipline of cancer neuroscience, an emerging discipline dedicated to exploring the relationship between cancer and nerves. The relationship and interconnection between cancer treatment and adverse reactions of the nervous system undoubtedly complements this field.

However, this study was conducted at the clinical research level, so specific information at the patient level, such as basic patient information, pathology reports, surgical conditions, and the time of NAEs start, could not be obtained, so further research is challenging. In addition, due to the small impact of most NAEs on the treatment and quality of life of patients, the evaluation of NAEs, especially non-serious neurological adverse reactions, is likely to be incomplete and inconsistent in clinical studies, which may also be one of the reasons for the large heterogeneity of the overall incidence of NAEs.

## Discussion and conclusion

In contrast to previous studies surrounding adverse events of PD-1/PD-L1 inhibitors that have focused mostly on high prevalence types, such as thyroid dysfunction and its systemic effects,^162^ this study provides new insights into the differences in the incidence of NAEs. The authors found that cancer patients treated with PD-1/PD-L1 inhibitors had a higher risk of serious compared with patients in the control group, and further explored the specific types of serious NAEs, which helps to establish the spectrum of immunotherapy-related neurotoxicity and provides relevant clinical information for the field of cancer immunotherapy.

This study is of great significance in both clinical and scientific research. In terms of clinical work, based on the summary of the incidence of NAEs in clinical trials, the authors have described the most comprehensive PD-1/PD-L1 neurologic toxicity map in existing studies. Summarizing the types of high-incidence NAEs and serious NAEs helps clinicians identify and take targeted inspections and interventions earlier when using related drugs, and strive for a better patients’ prognosis. In terms of basic research, previous studies have thoroughly explored the mechanism of action of PD-1/PD-L1 inhibitors,^163^ but there is still a gap in the study of immune-related adverse events, especially those of the NAEs. The analysis based on clinical patient data also provides some new ideas for research. Why the incidence of serious NAEs in immunotherapy is higher? Is there a connection between several types of neurotoxic events with high incidence, such as ischaemic stroke, with immunotherapy? Can the molecular mechanisms be further explored from these specific types of NRAEs?^164^ With the expansion of the scope of immunotherapy clinical trials, more patient-level NAEs data will also be obtained, making it possible to explore the relationship between NAEs and cancer prognosis, find biomarkers for early prediction of neurologic toxicity, and a series of related research contents.

Finally, this study explores the connection between clinical tumor immunotherapy and adverse effects on the nervous system. It opens up new opportunities for research in the interdisciplinary area of cancer neuroscience. Hopefully, further studies will be carried out to investigate this relationship and improve the treatment regimen and quality of life for cancer patients. Therefore, more efficient intervention measures could then be proposed to overcome the challenges of cancer.

## Abbreviations

PD-1, Programmed Death Receptor-1; PD-L1, Programmed Death Ligand-1; NAEs, Neurological Adverse Events; RCTs, Randomized Controlled Trials, RCT.

## Authors’ contributions

All authors contributed to the study's conception and design. Data collection and analysis were performed by Yanting Zhou, Hongyan Li and Hansong Yu. The first draft of the manuscript was written by Yanting Zhou and all authors commented on previous versions of the manuscript. All authors read and approved the final manuscript.

## Funding

This work was supported by the National Natural Science Foundation of China (NSFC) (grant number 82271373); the Natural Science Foundation of Beijing Municipality (Beijing Natural Science Foundation) (grant number 7232075).

## Declaration of competing interest

The authors declare no conflicts of interest.

## Appendix Supplementary materials

Image, application 1
