# Supplementary material for: Neurological adverse events of PD-1/PD-L1 immune checkpoint inhibitors in clinical trials: A meta-analysis
Source: Clinics (Sao Paulo). 2025 Jul 10;80:100698. doi: 10.1016/j.clinsp.2025.100698 (PMC12275200; doi:10.1016/j.clinsp.2025.100698)
Supplement: Supplementary file 1 [file mmc1.docx]

**Neurological adverse events of PD-1/PD-L1 immune checkpoint inhibitors in clinical trials: A meta-analysis**

Yanting Zhou, MM^1^; Hansong Yu, MM^1^; Hongyan Li, PhD, MD^1*^

1 General Surgery Department, Xuanwu Hospital, Beijing 100053, China

E-mail: [hongyanli@xwhosp.org](mailto:hongyanli@xwhosp.org)

**Supplement**

**eTable 1. PRISMA Standard**

**eTable 2.** **Baseline characteristics of publications and patients**

**eTable 3. Risk of Bias of Included Publications**

**eFigure 1. AMSTAR Standard**

**eFigure 2. Forest plots of the incidence of NAEs**

**eMethods. Search strategy**

**eTable 1. PRISMA Standard**

| Section and Topic | Item | Checklist item | Location where item is reported |
| --- | --- | --- | --- |
| TITLE | | |  |
| Title | 1 | Identify the report as a systematic review. | Page1 |
| ABSTRACT | | |  |
| Abstract | 2 | See the PRISMA 2020 for Abstracts checklist. | Page2 |
| INTRODUCTION | | |  |
| Rationale | 3 | Describe the rationale for the review in the context of existing knowledge. | Page3 |
| Objectives | 4 | Provide an explicit statement of the objective(s) or question(s) the review addresses. | Page3 |
| METHODS | | |  |
| Eligibility criteria | 5 | Specify the inclusion and exclusion criteria for the review and how studies were grouped for the syntheses. | Page4-6 |
| Information sources | 6 | Specify all databases, registers, websites, organisations, reference lists and other sources searched or consulted to identify studies. Specify the date when each source was last searched or consulted. | Page4-6 |
| Search strategy | 7 | Present the full search strategies for all databases, registers and websites, including any filters and limits used. | Page4-6 |
| Selection process | 8 | Specify the methods used to decide whether a study met the inclusion criteria of the review, including how many reviewers screened each record and each report retrieved, whether they worked independently, and if applicable, details of automation tools used in the process. | Page4-6 |
| Data collection process | 9 | Specify the methods used to collect data from reports, including how many reviewers collected data from each report, whether they worked independently, any processes for obtaining or confirming data from study investigators, and if applicable, details of automation tools used in the process. | Page6-7 |
| Data items | 10a | List and define all outcomes for which data were sought. Specify whether all results that were compatible with each outcome domain in each study were sought (e.g. for all measures, time points, analyses), and if not, the methods used to decide which results to collect. | Page3-4 |
|  | 10b | List and define all other variables for which data were sought (e.g. participant and intervention characteristics, funding sources). Describe any assumptions made about any missing or unclear information. | Page2-3 |
| Study risk of bias assessment | 11 | Specify the methods used to assess risk of bias in the included studies, including details of the tool(s) used, how many reviewers assessed each study and whether they worked independently, and if applicable, details of automation tools used in the process. | Page2-3 |
| Effect measures | 12 | Specify for each outcome the effect measure(s) (e.g. risk ratio, mean difference) used in the synthesis or presentation of results. | Page2-3 |
| Synthesis methods | 13a | Describe the processes used to decide which studies were eligible for each synthesis (e.g. tabulating the study intervention characteristics and comparing against the planned groups for each synthesis (item #5)). | Page4-5 |
|  | 13b | Describe any methods required to prepare the data for presentation or synthesis, such as handling of missing summary statistics, or data conversions. | Page4-5 |
|  | 13c | Describe any methods used to tabulate or visually display results of individual studies and syntheses. | Page4-5 |
|  | 13d | Describe any methods used to synthesize results and provide a rationale for the choice(s). If meta-analysis was performed, describe the model(s), method(s) to identify the presence and extent of statistical heterogeneity, and software package(s) used. | Page4-5 |
|  | 13e | Describe any methods used to explore possible causes of heterogeneity among study results (e.g. subgroup analysis, meta-regression). | Page4-5 |
|  | 13f | Describe any sensitivity analyses conducted to assess robustness of the synthesized results. | Page4-5 |
| Reporting bias assessment | 14 | Describe any methods used to assess risk of bias due to missing results in a synthesis (arising from reporting biases). | Page5 |
| Certainty assessment | 15 | Describe any methods used to assess certainty (or confidence) in the body of evidence for an outcome. | Page4 |
| RESULTS | | |  |
| Study selection | 16a | Describe the results of the search and selection process, from the number of records identified in the search to the number of studies included in the review, ideally using a flow diagram. | Page5-6 |
|  | 16b | Cite studies that might appear to meet the inclusion criteria, but which were excluded, and explain why they were excluded. | Page5-6 |
| Study characteristics | 17 | Cite each included study and present its characteristics. | Page5 |
| Risk of bias in studies | 18 | Present assessments of risk of bias for each included study. | Page7 |
| Results of individual studies | 19 | For all outcomes, present, for each study: (a) summary statistics for each group (where appropriate) and (b) an effect estimate and its precision (e.g. confidence/credible interval), ideally using structured tables or plots. | Page5-7 |
| Results of syntheses | 20a | For each synthesis, briefly summarise the characteristics and risk of bias among contributing studies. | Page6-7 |
|  | 20b | Present results of all statistical syntheses conducted. If meta-analysis was done, present for each the summary estimate and its precision (e.g. confidence/credible interval) and measures of statistical heterogeneity. If comparing groups, describe the direction of the effect. | Page6-7 |
|  | 20c | Present results of all investigations of possible causes of heterogeneity among study results. | Page6-7 |
|  | 20d | Present results of all sensitivity analyses conducted to assess the robustness of the synthesized results. | Page6-7 |
| Reporting biases | 21 | Present assessments of risk of bias due to missing results (arising from reporting biases) for each synthesis assessed. | Supplements |
| Certainty of evidence | 22 | Present assessments of certainty (or confidence) in the body of evidence for each outcome assessed. | Page6-7 |
| DISCUSSION | | |  |
| Discussion | 23a | Provide a general interpretation of the results in the context of other evidence. | Page12-13 |
|  | 23b | Discuss any limitations of the evidence included in the review. | Page12-13 |
|  | 23c | Discuss any limitations of the review processes used. | Page12-13 |
|  | 23d | Discuss implications of the results for practice, policy, and future research. | Page12-13 |
| OTHER INFORMATION | | |  |
| Registration and protocol | 24a | Provide registration information for the review, including register name and registration number, or state that the review was not registered. | Page4 |
|  | 24b | Indicate where the review protocol can be accessed, or state that a protocol was not prepared. | Page4 |
|  | 24c | Describe and explain any amendments to information provided at registration or in the protocol. | Page4 |
| Support | 25 | Describe sources of financial or non-financial support for the review, and the role of the funders or sponsors in the review. | Page10 |
| Competing interests | 26 | Declare any competing interests of review authors. | Page42 |
| Availability of data, code and other materials | 27 | Report which of the following are publicly available and where they can be found: template data collection forms; data extracted from included studies; data used for all analyses; analytic code; any other materials used in the review. | Page42 |

**
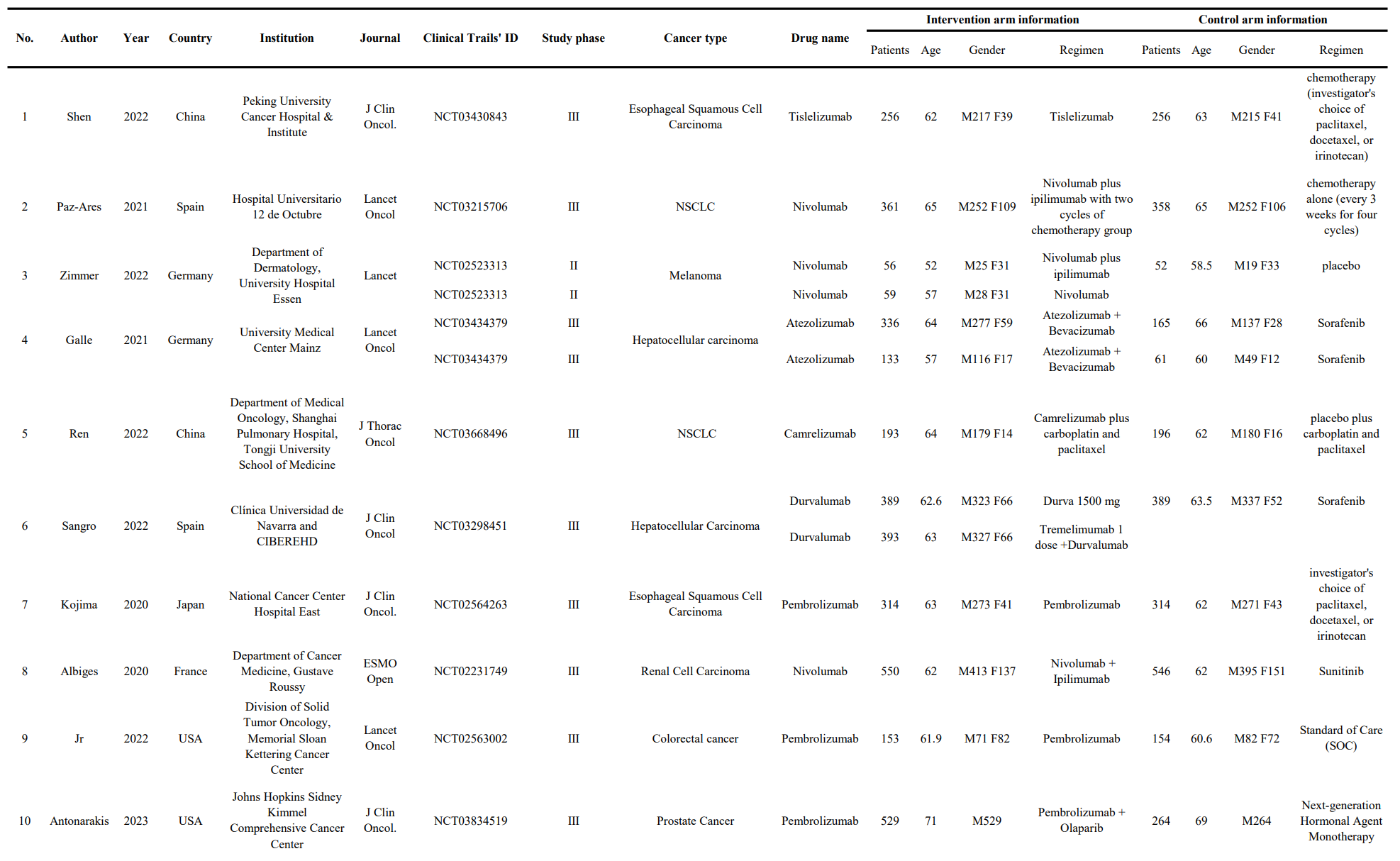
 eTable 2. Baseline characteristics of publications and patients（An excel file submitted also）**

**
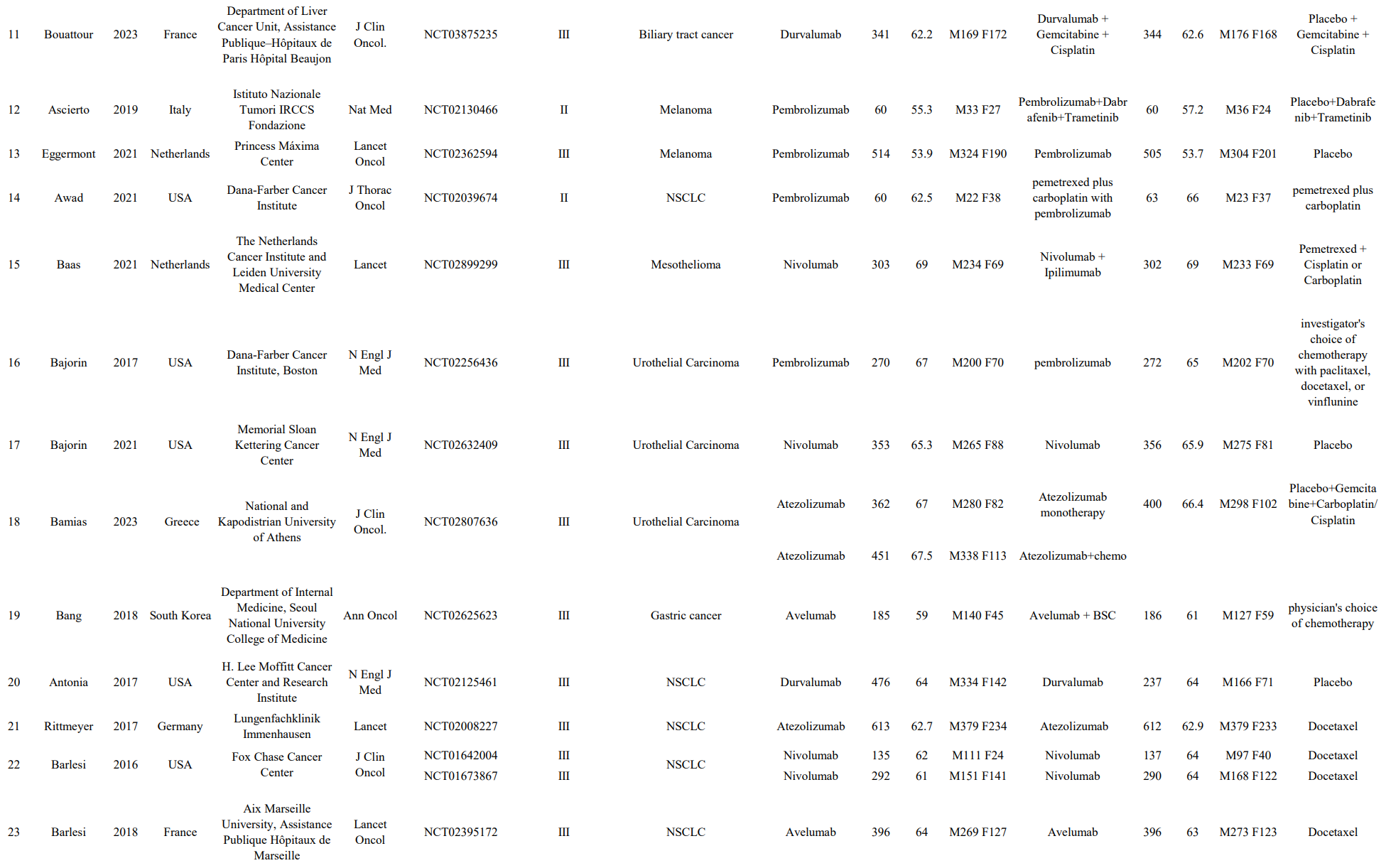
**

**
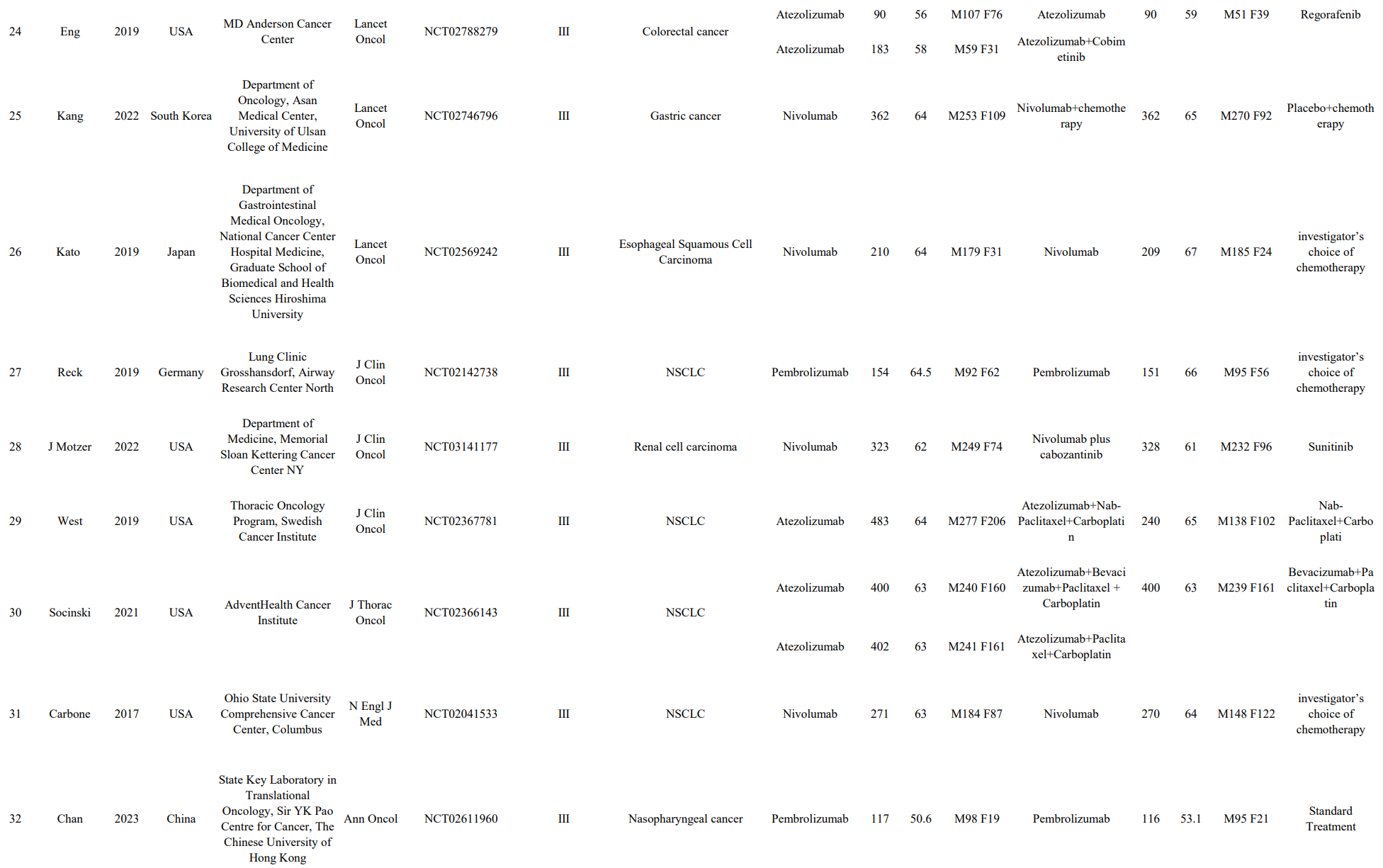
**

**
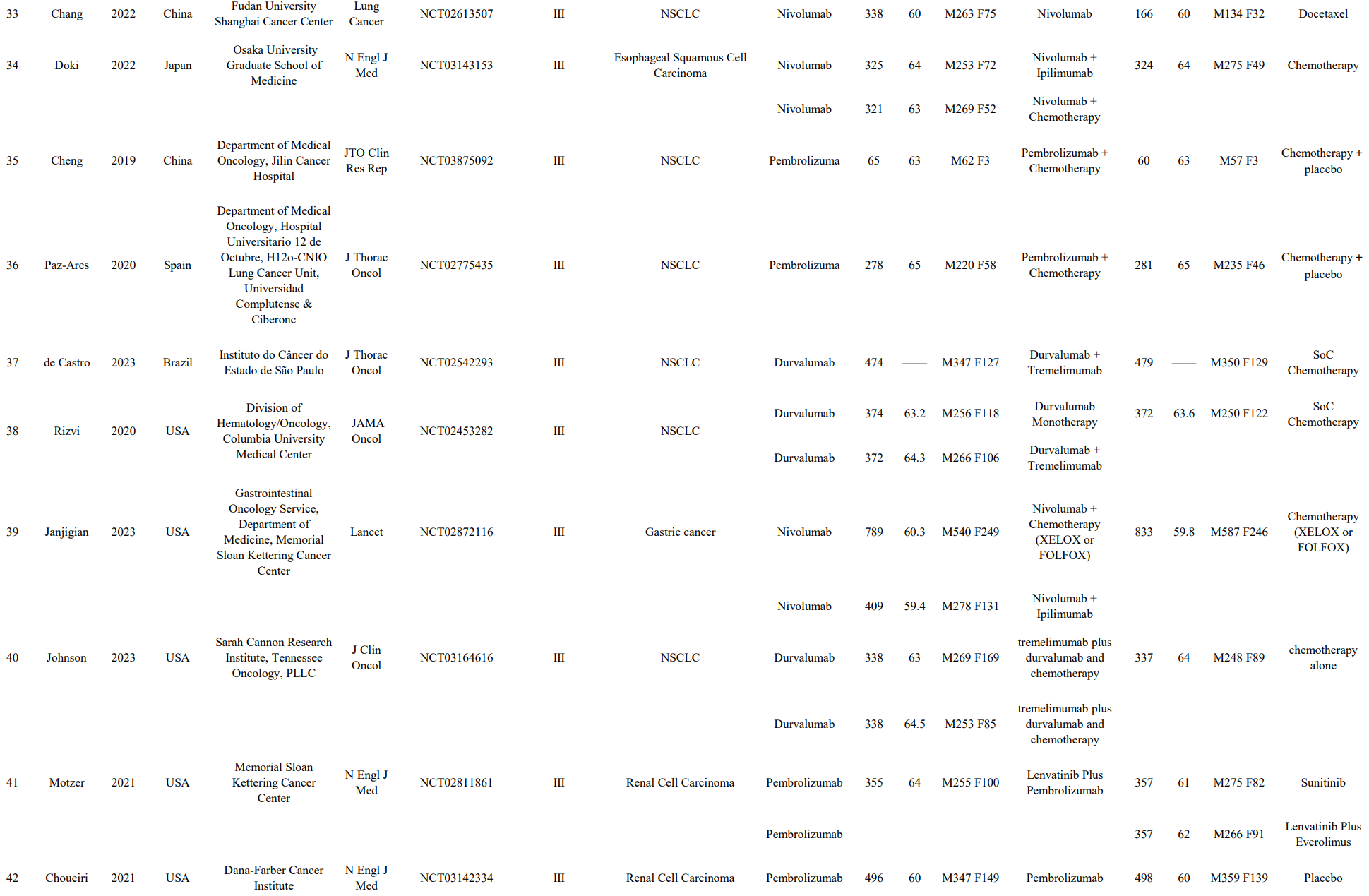
**

**
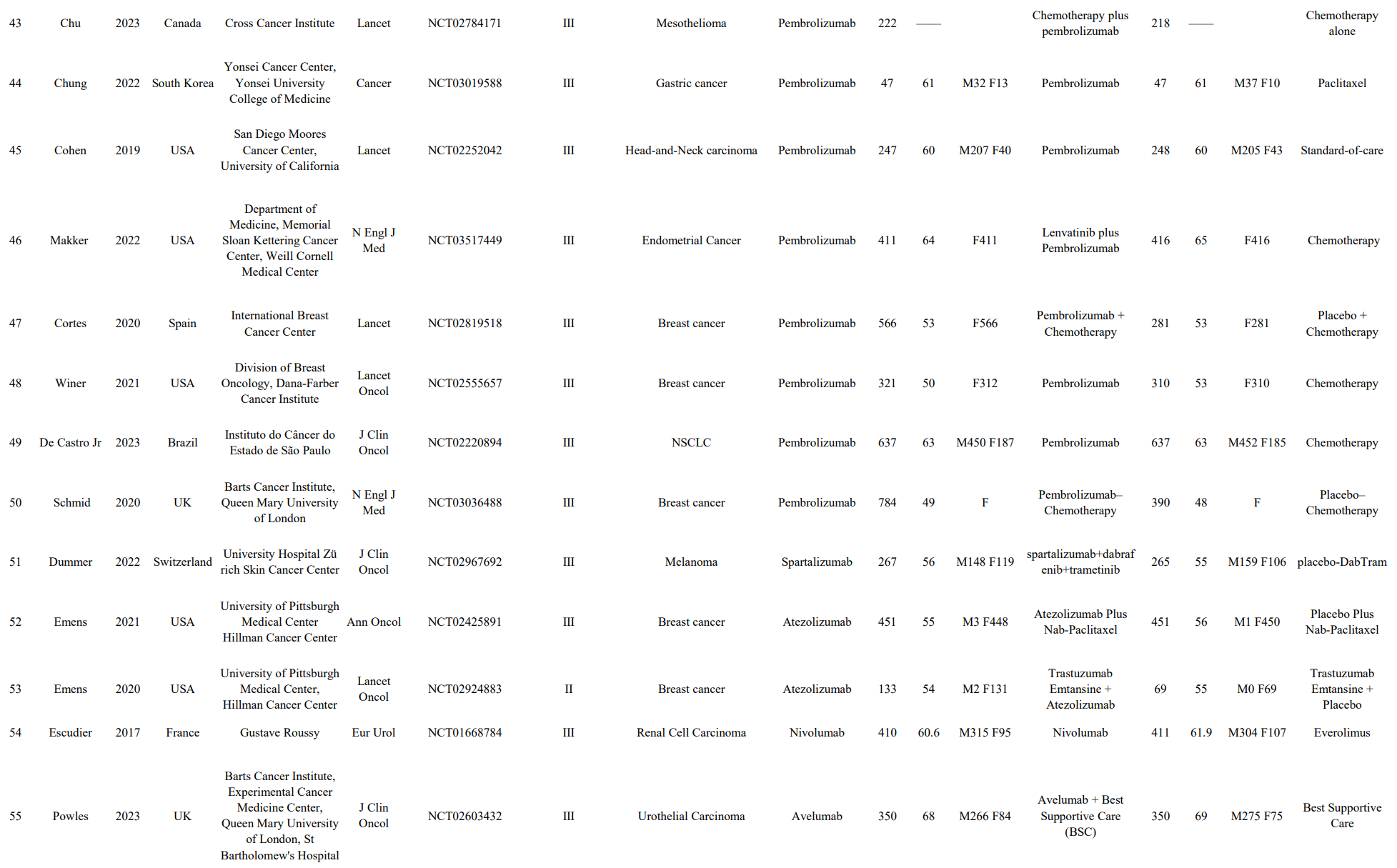
**

**
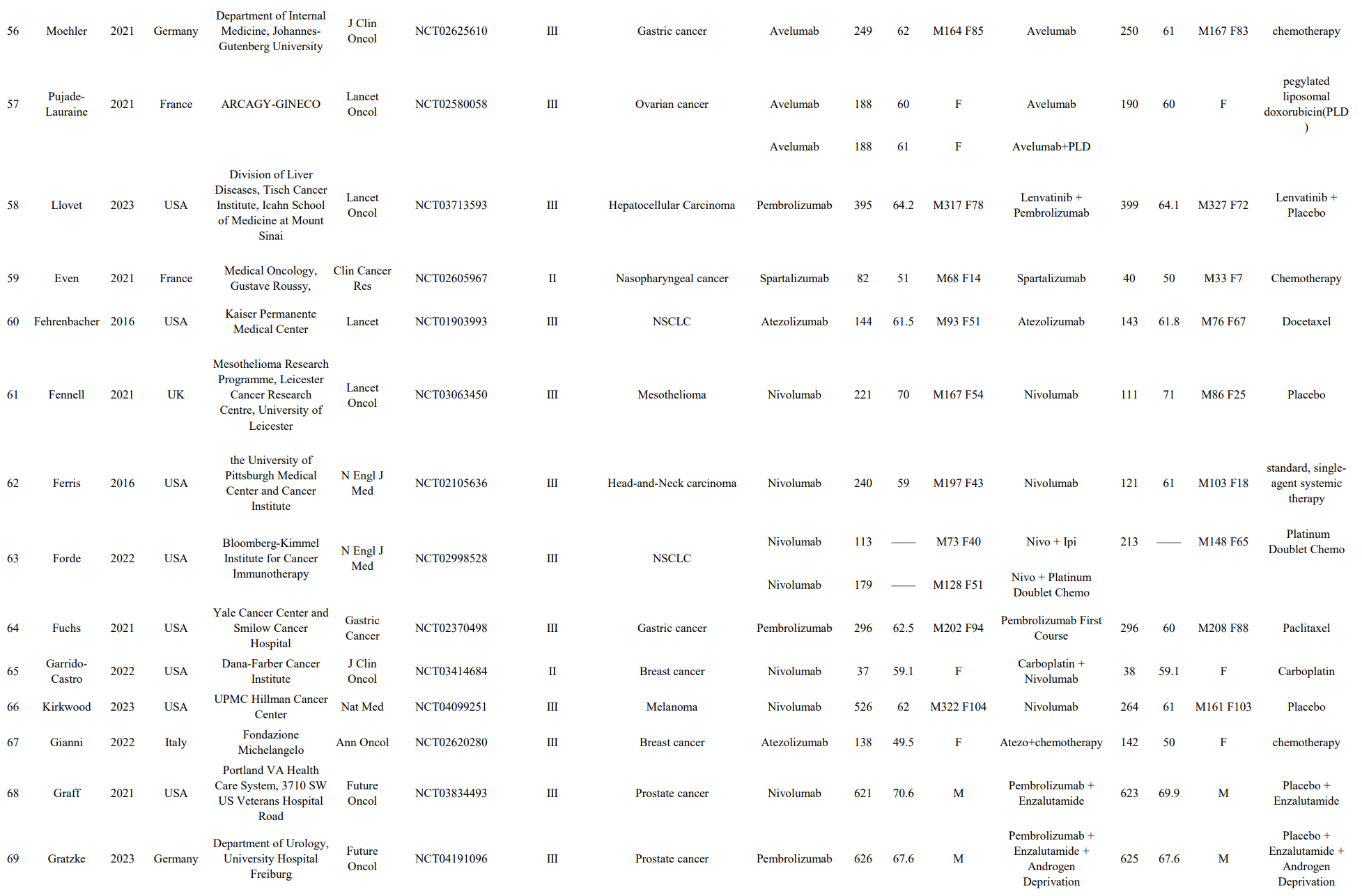
**

**
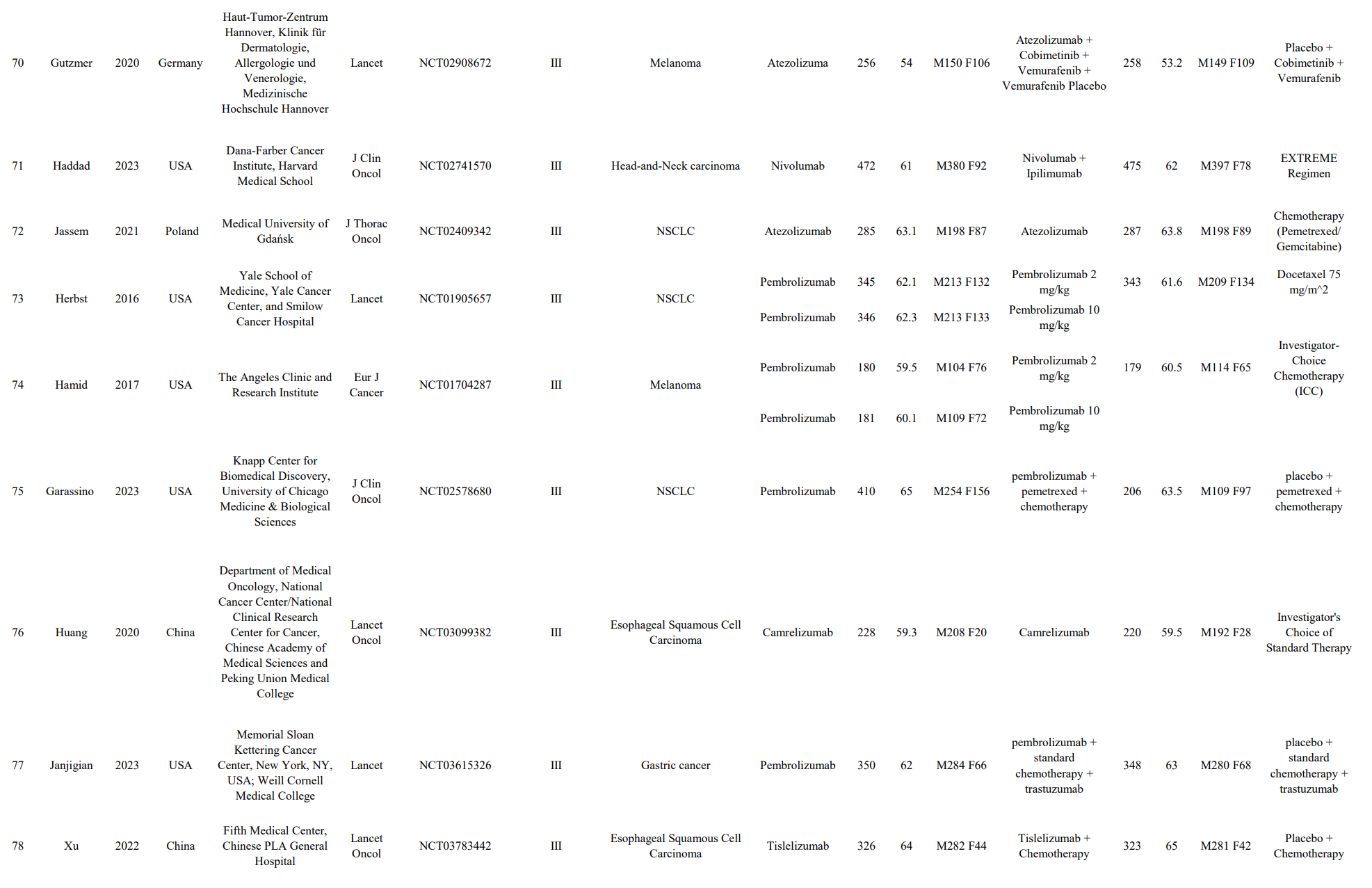
**

**
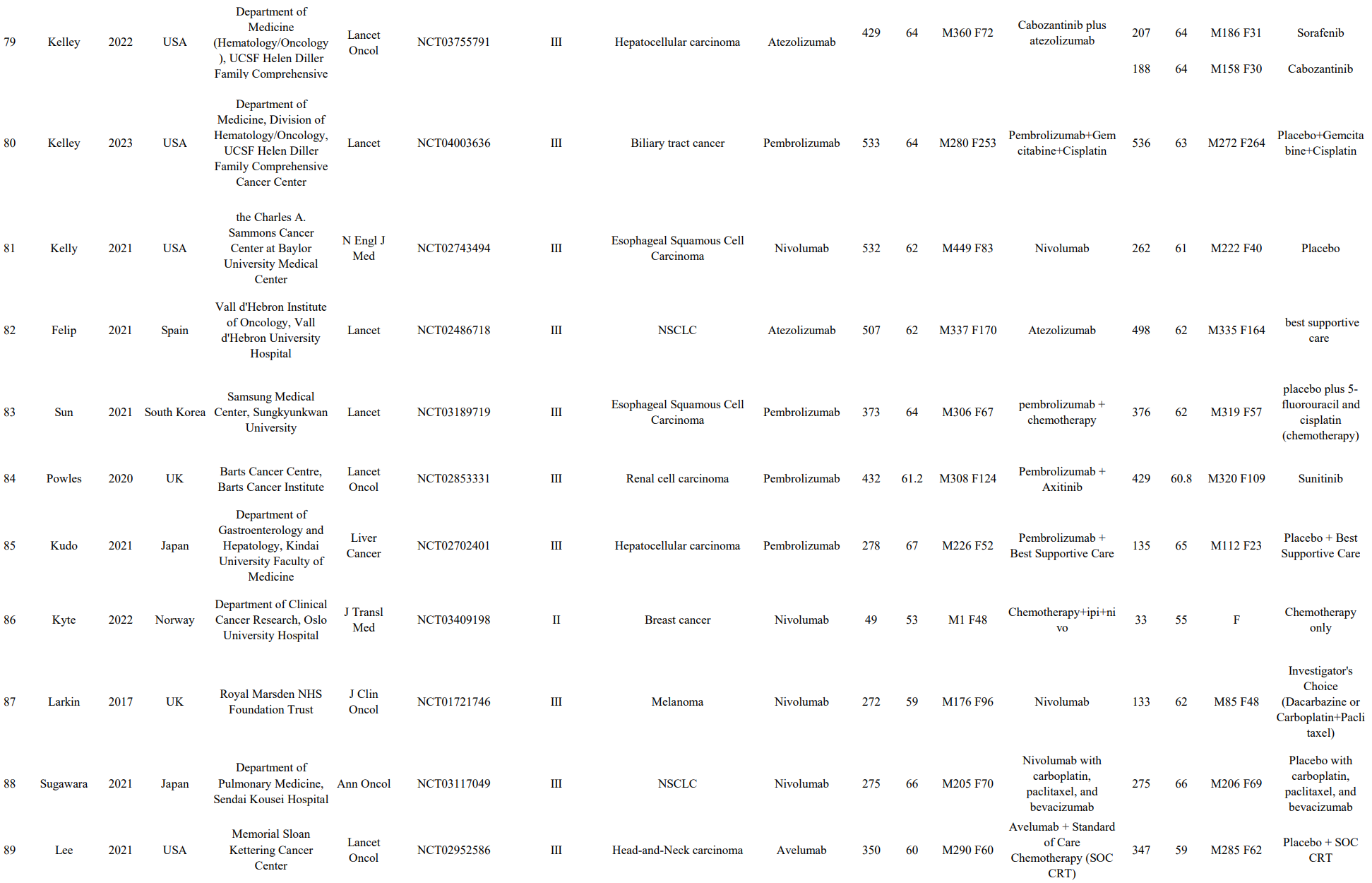
**

**
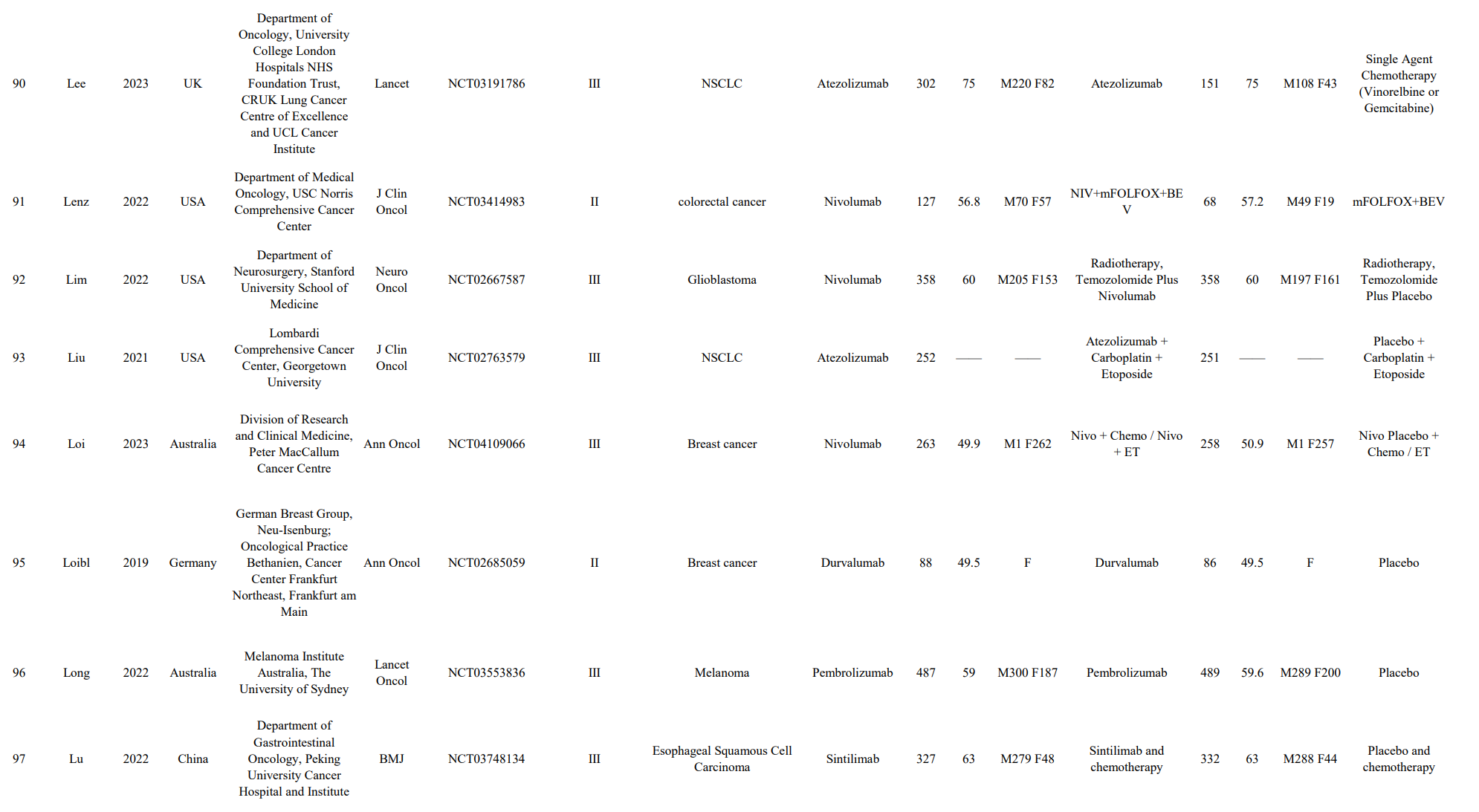
**

**
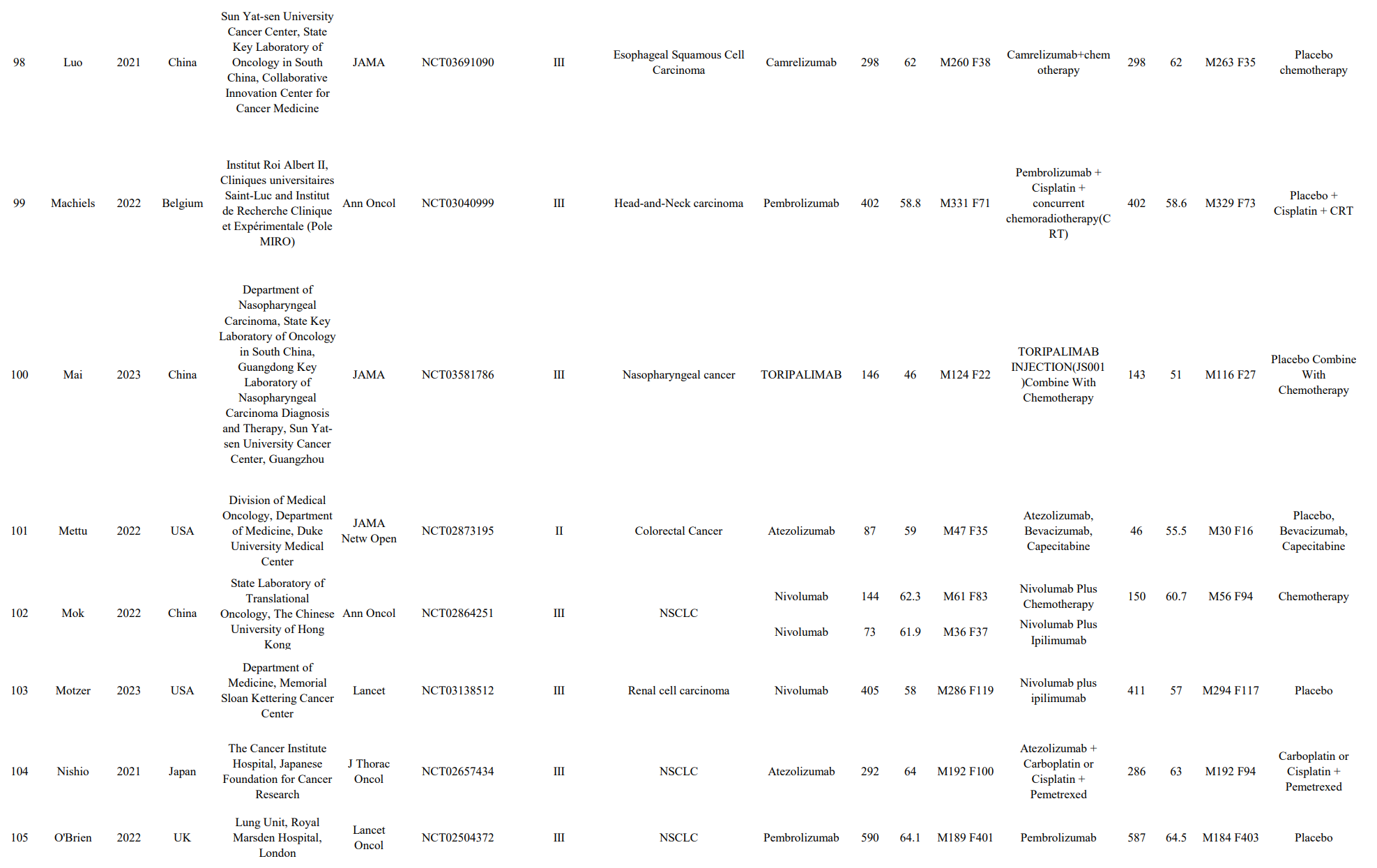
**

**
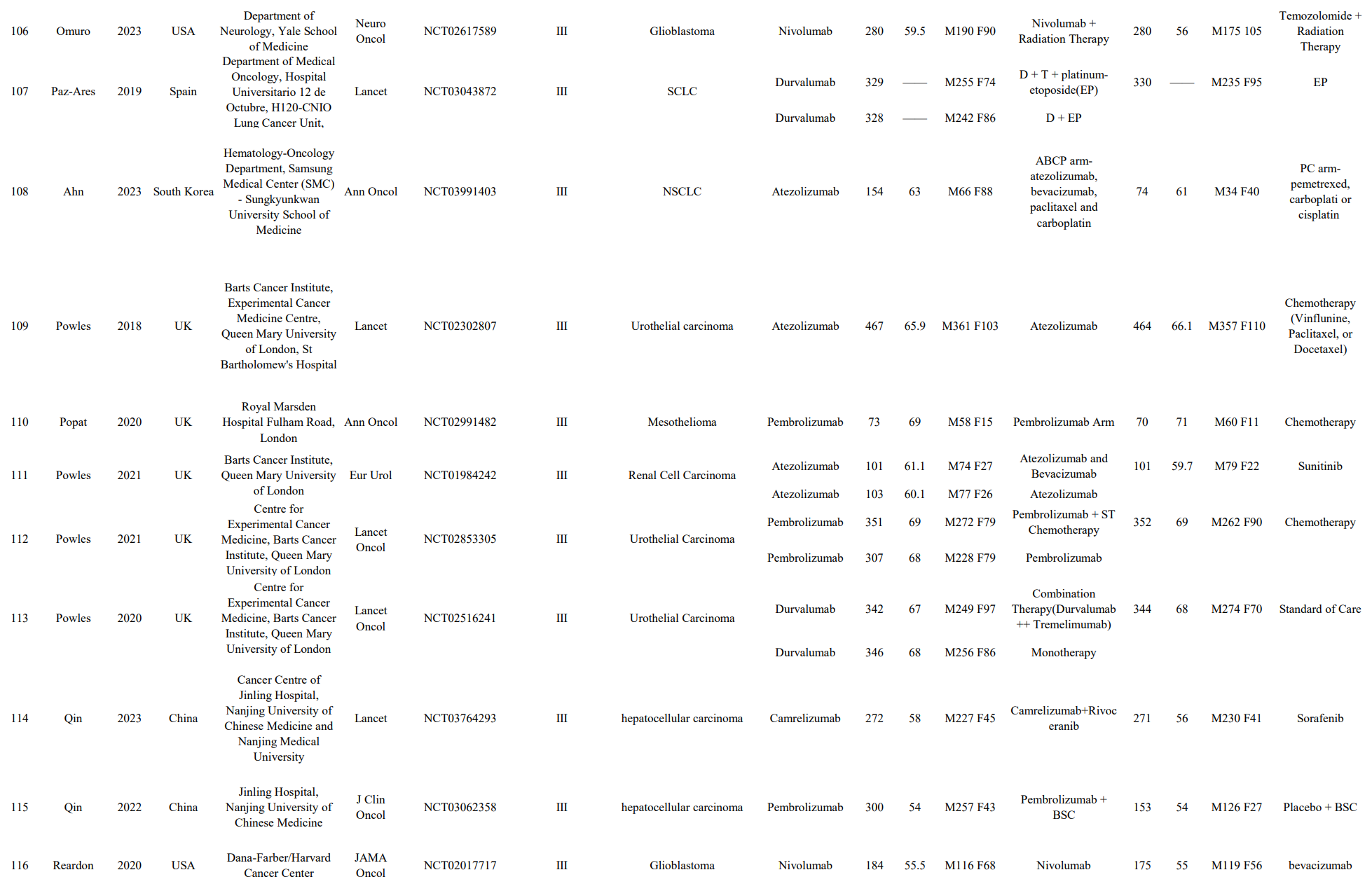
**

**
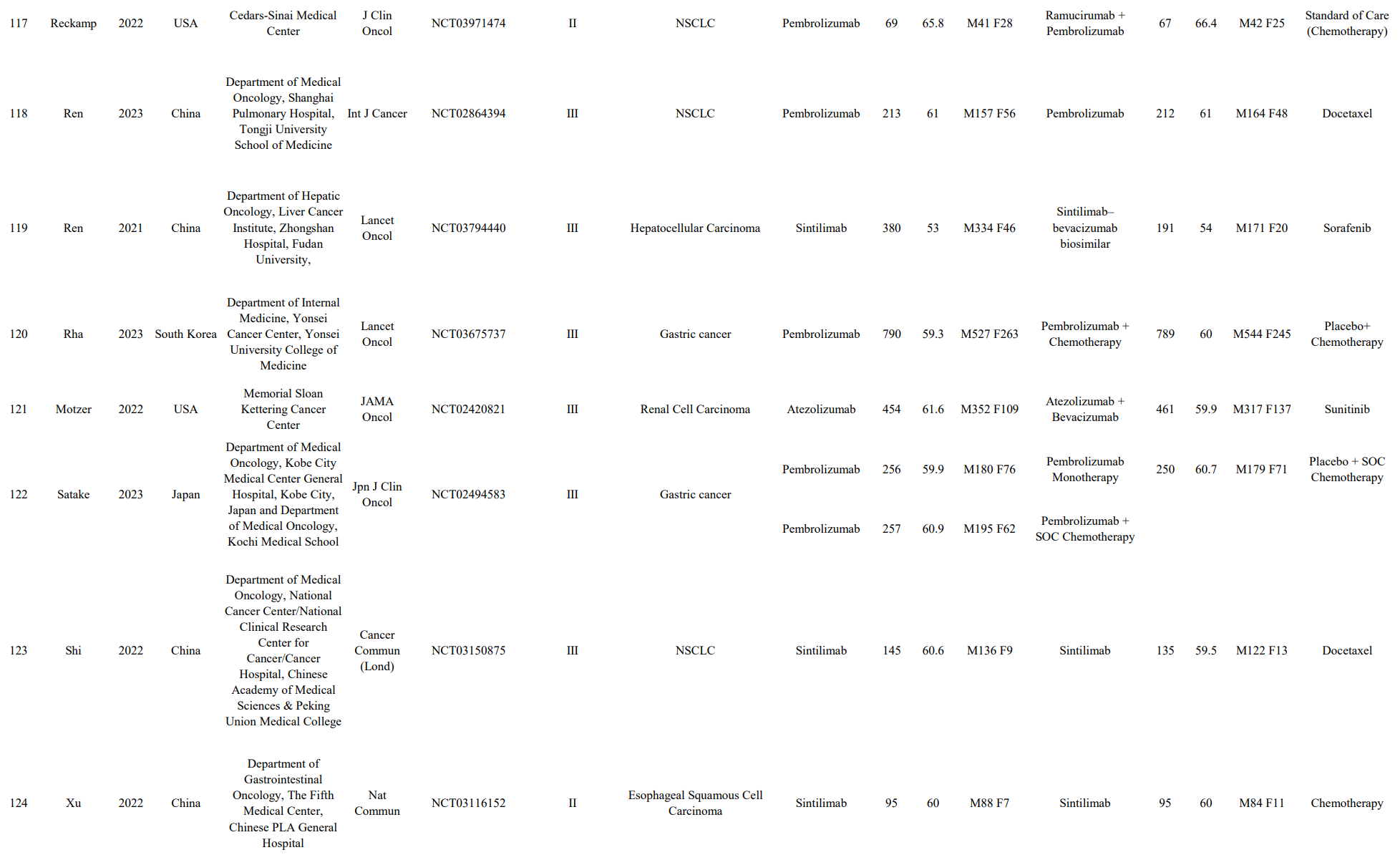
**

**
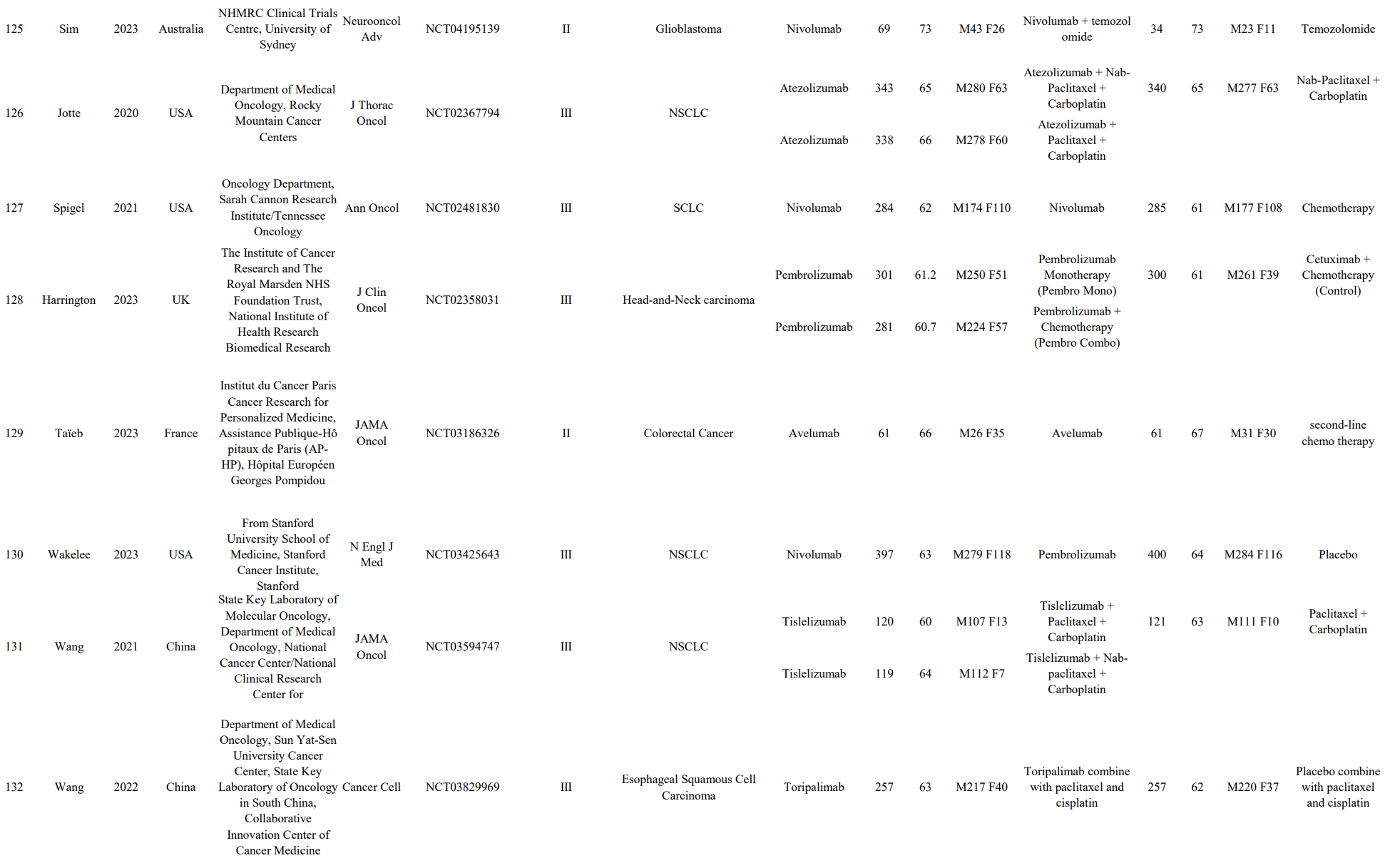
**

**
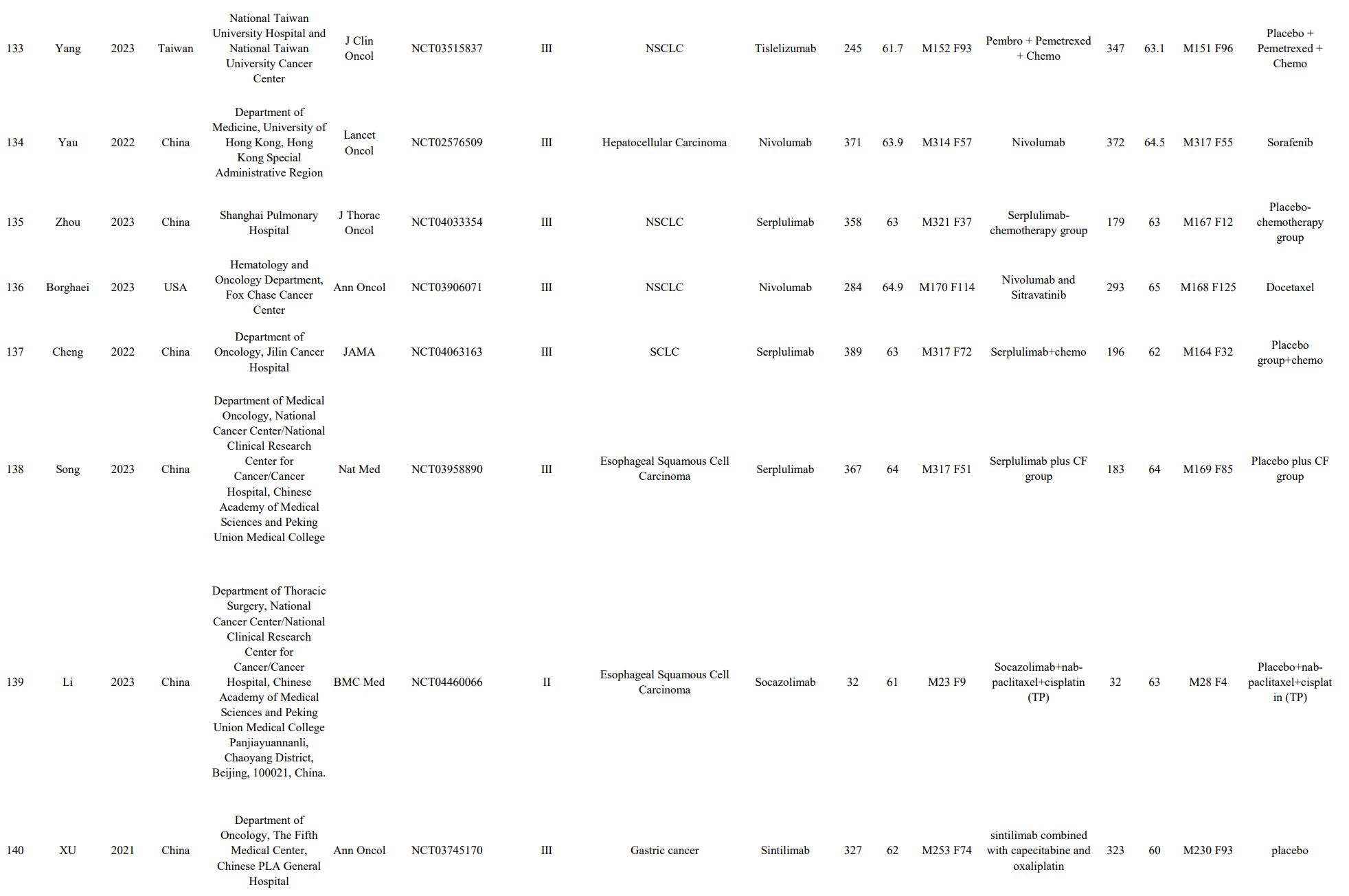
**

**
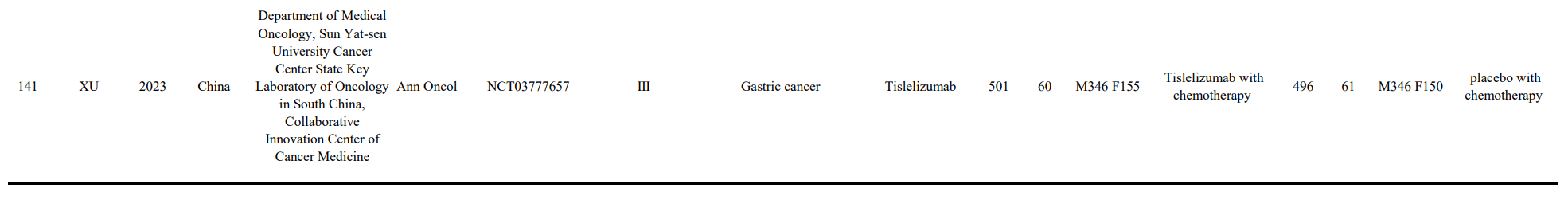
**

**eTable 3. Risk of Bias of Included Publications**

| **No.** | **Author** | **Year** | **Randomization** | **Allocation  Concealment** | **Blinding of Participants and stuff** | **Blinding of Outcome Assessors** | **Incomplete Outcome Data** | **Selective Outcome Reporting** | **Other Sources of Bias** |
| --- | --- | --- | --- | --- | --- | --- | --- | --- | --- |
| 1 | Shen | 2022 | Low | Low | Low | Low | Low | Low | Low |
| 2 | Paz-Ares | 2021 | Low | Low | Low | Low | Low | Low | Low |
| 3 | Zimmer | 2022 | Low | Low | Low | Low | Low | High | Low |
| 4 | Galle | 2021 | Low | Low | Low | Low | Low | Low | Low |
| 5 | Ren | 2022 | Low | Low | Low | Low | Low | High | Low |
| 6 | Sangro | 2022 | Low | Low | Low | Low | Low | Low | Low |
| 7 | Kojima | 2020 | Low | Low | Low | Low | Low | Low | Low |
| 8 | Albiges | 2020 | Low | Low | Low | Low | Low | Low | Low |
| 9 | Jr | 2022 | Low | Low | Low | Low | Low | Low | Low |
| 10 | Antonarakis | 2023 | Low | Low | Low | Low | Low | Low | Low |
| 11 | Bouattour | 2023 | Low | Low | Low | Low | Low | Low | Low |
| 12 | Ascierto | 2019 | Low | Low | Low | Low | Low | Low | Low |
| 13 | Eggermont | 2021 | Low | Low | Low | Low | Low | Low | Low |
| 14 | Awad | 2021 | Low | Low | Low | Low | Low | Low | Low |
| 15 | Baas | 2021 | Low | Low | Low | Low | Low | Low | Low |
| 16 | Bajorin | 2017 | Low | Low | Low | Low | Low | Low | Low |
| 17 | Bajorin | 2021 | Low | Low | Low | Low | Low | Low | Low |
| 18 | Bamias | 2023 | Low | Low | Low | Low | Low | Low | Low |
| 19 | Bang | 2018 | Low | Low | Low | Low | Low | Low | Low |
| 20 | Antonia | 2017 | Low | Low | Low | Low | Low | Low | Low |
| 21 | Rittmeyer | 2017 | Low | Low | Low | Low | Low | Low | Low |
| 22 | Barlesi | 2016 | Low | Low | Low | Low | Low | Low | Low |
| 23 | Barlesi | 2018 | Low | Low | Low | Low | Low | Low | Low |
| 24 | Eng | 2019 | Low | Low | Low | Low | Low | Low | Low |
| 25 | Kang | 2022 | Low | Low | Low | Low | Low | High | Low |
| 26 | Kato | 2019 | Low | Low | Low | Low | Low | Low | Low |
| 27 | Reck | 2019 | Low | Low | Low | Low | Low | Low | Low |
| 28 | J Motzer | 2022 | Low | Low | Low | Low | Low | Low | Low |
| 29 | West | 2019 | Low | Low | Low | Low | Low | Low | Low |
| 30 | Socinski | 2021 | Low | Low | Low | Low | Low | Low | Low |
| 31 | Carbone | 2017 | Low | Low | Low | Low | Low | Low | Low |
| 32 | Chan | 2023 | Low | Low | Low | Low | Low | Low | Low |
| 33 | Chang | 2022 | Low | Low | Low | Low | Low | Low | Low |
| 34 | Doki | 2022 | Low | Low | Low | Low | Low | Low | Low |
| 35 | Cheng | 2019 | Low | Low | Low | Low | Low | Low | Low |
| 36 | Paz-Ares | 2020 | Low | Low | Low | Low | Low | Low | Low |
| 37 | de Castro | 2023 | Low | Low | Low | Low | High | Low | Low |
| 38 | Rizvi | 2020 | Low | Low | Low | Low | Low | Low | Low |
| 39 | Janjigian | 2023 | Low | Low | Low | Low | Low | Low | Low |
| 40 | Johnson | 2023 | Low | Low | Low | Low | Low | Low | Low |
| 41 | Motzer | 2021 | Low | Low | Low | Low | Low | Low | Low |
| 42 | Choueiri | 2021 | Low | Low | Low | Low | Low | Low | Low |
| 43 | Chu | 2023 | Low | Low | Low | Low | High | High | Low |
| 44 | Chung | 2022 | Low | Low | Low | Low | Low | Low | Low |
| 45 | Cohen | 2019 | Low | Low | Low | Low | Low | Low | Low |
| 46 | Makker | 2022 | Low | Low | Low | Low | Low | Low | Low |
| 47 | Cortes | 2020 | Low | Low | Low | Low | Low | Low | Low |
| 48 | Winer | 2021 | Low | Low | Low | Low | Low | Low | Low |
| 49 | De Castro Jr | 2023 | Low | Low | Low | Low | Low | Low | Low |
| 50 | Schmid | 2020 | Low | Low | Low | Low | Low | High | Low |
| 51 | Dummer | 2022 | Low | Low | Low | Low | Low | Low | Low |
| 52 | Emens | 2021 | Low | Low | Low | Low | Low | Low | Low |
| 53 | Emens | 2020 | Low | Low | Low | Low | Low | Low | Low |
| 54 | Escudier | 2017 | Low | Low | Low | Low | Low | Low | Low |
| 55 | Powles | 2023 | Low | Low | Low | Low | Low | Low | Low |
| 56 | Moehler | 2021 | Low | Low | Low | Low | Low | Low | Low |
| 57 | Pujade-Lauraine | 2021 | Low | Low | Low | Low | Low | Low | Low |
| 58 | Llovet | 2023 | Low | Low | Low | Low | Low | Low | Low |
| 59 | Even | 2021 | Low | Low | Low | Low | Low | Low | Low |
| 60 | Fehrenbacher | 2016 | Low | Low | Low | Low | Low | Low | Low |
| 61 | Fennell | 2021 | Low | Low | Low | Low | Low | Low | Low |
| 62 | Ferris | 2016 | Low | Low | Low | Low | Low | Low | Low |
| 63 | Forde | 2022 | Low | Low | Low | Low | High | Low | Low |
| 64 | Fuchs | 2021 | Low | Low | Low | Low | Low | Low | Low |
| 65 | Garrido-Castro | 2022 | Low | Low | Low | Low | Low | Low | Low |
| 66 | Kirkwood | 2023 | Low | Low | Low | Low | Low | Low | Low |
| 67 | Gianni | 2022 | Low | Low | Low | Low | Low | Low | Low |
| 68 | Graff | 2021 | Low | Low | Low | Low | Low | Low | Low |
| 69 | Gratzke | 2023 | Low | Low | Low | Low | Low | Low | Low |
| 70 | Gutzmer | 2020 | Low | Low | Low | Low | Low | Low | Low |
| 71 | Haddad | 2023 | Low | Low | Low | Low | Low | Low | Low |
| 72 | Jassem | 2021 | Low | Low | Low | Low | Low | Low | Low |
| 73 | Herbst | 2016 | Low | Low | Low | Low | Low | Low | Low |
| 74 | Hamid | 2017 | Low | Low | Low | Low | Low | Low | Low |
| 75 | Garassino | 2023 | Low | Low | Low | Low | Low | Low | Low |
| 76 | Huang | 2020 | Low | Low | Low | Low | Low | Low | Low |
| 77 | Janjigian | 2023 | Low | Low | Low | Low | Low | High | Low |
| 78 | Xu | 2022 | Low | Low | Low | Low | Low | Low | Low |
| 79 | Kelley | 2022 | Low | Low | Low | Low | Low | High | Low |
| 80 | Kelley | 2023 | Low | Low | Low | Low | Low | Low | Low |
| 81 | Kelly | 2021 | Low | Low | Low | Low | Low | Low | Low |
| 82 | Felip | 2021 | Low | Low | Low | Low | Low | Low | Low |
| 83 | Sun | 2021 | Low | Low | Low | Low | Low | Low | Low |
| 84 | Powles | 2020 | Low | Low | Low | Low | Low | Low | Low |
| 85 | Kudo | 2021 | Low | Low | Low | Low | Low | Low | Low |
| 86 | Kyte | 2022 | Low | Low | Low | Low | Low | High | Low |
| 87 | Larkin | 2017 | Low | Low | Low | Low | Low | Low | Low |
| 88 | Sugawara | 2021 | Low | Low | Low | Low | Low | Low | Low |
| 89 | Lee | 2021 | Low | Low | Low | Low | Low | Low | Low |
| 90 | Lee | 2023 | Low | Low | Low | Low | Low | Low | Low |
| 91 | Lenz | 2022 | Low | Low | Low | Low | Low | Low | Low |
| 92 | Lim | 2022 | Low | Low | Low | Low | Low | Low | Low |
| 93 | Liu | 2021 | Low | Low | Low | Low | High | Low | Low |
| 94 | Loi | 2023 | Low | Low | Low | Low | Low | Low | Low |
| 95 | Loibl | 2019 | Low | Low | Low | Low | Low | High | Low |
| 96 | Long | 2022 | Low | Low | Low | Low | Low | Low | Low |
| 97 | Lu | 2022 | Low | Low | Low | Low | Low | High | Low |
| 98 | Luo | 2021 | Low | Low | Low | Low | Low | High | Low |
| 99 | Machiels | 2022 | Low | Low | Low | Low | Low | Low | Low |
| 100 | Mai | 2023 | Low | Low | Low | Low | Low | Low | Low |
| 101 | Mettu | 2022 | Low | Low | Low | Low | Low | Low | Low |
| 102 | Mok | 2022 | Low | Low | Low | Low | Low | Low | Low |
| 103 | Motzer | 2023 | Low | Low | Low | Low | Low | Low | Low |
| 104 | Nishio | 2021 | Low | Low | Low | Low | Low | Low | Low |
| 105 | O'Brien | 2022 | Low | Low | Low | Low | Low | Low | Low |
| 106 | Omuro | 2023 | Low | Low | Low | Low | Low | Low | Low |
| 107 | Paz-Ares | 2019 | Low | Low | Low | Low | High | Low | Low |
| 108 | Ahn | 2023 | Low | Low | Low | Low | Low | High | Low |
| 109 | Powles | 2018 | Low | Low | Low | Low | Low | Low | Low |
| 110 | Popat | 2020 | Low | Low | Low | Low | Low | Low | Low |
| 111 | Powles | 2021 | Low | Low | Low | Low | Low | Low | Low |
| 112 | Powles | 2021 | Low | Low | Low | Low | Low | Low | Low |
| 113 | Powles | 2020 | Low | Low | Low | Low | Low | Low | Low |
| 114 | Qin | 2023 | Low | Low | Low | Low | Low | Low | Low |
| 115 | Qin | 2022 | Low | Low | Low | Low | Low | Low | Low |
| 116 | Reardon | 2020 | Low | Low | Low | Low | Low | Low | Low |
| 117 | Reckamp | 2022 | Low | Low | Low | Low | Low | Low | Low |
| 118 | Ren | 2023 | Low | Low | Low | Low | Low | Low | Low |
| 119 | Ren | 2021 | Low | Low | Low | Low | Low | High | Low |
| 120 | Rha | 2023 | Low | Low | Low | Low | Low | Low | Low |
| 121 | Motzer | 2022 | Low | Low | Low | Low | Low | Low | Low |
| 122 | Satake | 2023 | Low | Low | Low | Low | Low | Low | Low |
| 123 | Shi | 2022 | Low | Low | Low | Low | Low | Low | Low |
| 124 | Xu | 2022 | Low | Low | Low | Low | Low | Low | Low |
| 125 | Sim | 2023 | Low | Low | Low | Low | Low | Low | Low |
| 126 | Jotte | 2020 | Low | Low | Low | Low | Low | Low | Low |
| 127 | Spigel | 2021 | Low | Low | Low | Low | Low | Low | Low |
| 128 | Harrington | 2023 | Low | Low | Low | Low | Low | Low | Low |
| 129 | Taïeb | 2023 | Low | Low | Low | Low | Low | High | Low |
| 130 | Wakelee | 2023 | Low | Low | Low | Low | Low | High | Low |
| 131 | Wang | 2021 | Low | Low | Low | Low | Low | Low | Low |
| 132 | Wang | 2022 | Low | Low | Low | Low | Low | Low | Low |
| 133 | Yang | 2023 | Low | Low | Low | Low | Low | Low | Low |
| 134 | Yau | 2022 | Low | Low | Low | Low | Low | Low | Low |
| 135 | Zhou | 2023 | Low | Low | Low | Low | Low | High | Low |
| 136 | Borghaei | 2023 | Low | Low | Low | Low | Low | Low | Low |
| 137 | Cheng | 2022 | Low | Low | Low | Low | Low | High | Low |
| 138 | Song | 2023 | Low | Low | Low | Low | Low | High | Low |
| 139 | Li | 2023 | Low | Low | Low | Low | Low | High | Low |
| 140 | XU | 2021 | Low | Low | Low | Low | Low | High | Low |
| 141 | XU | 2023 | Low | Low | Low | Low | Low | High | Low |

**
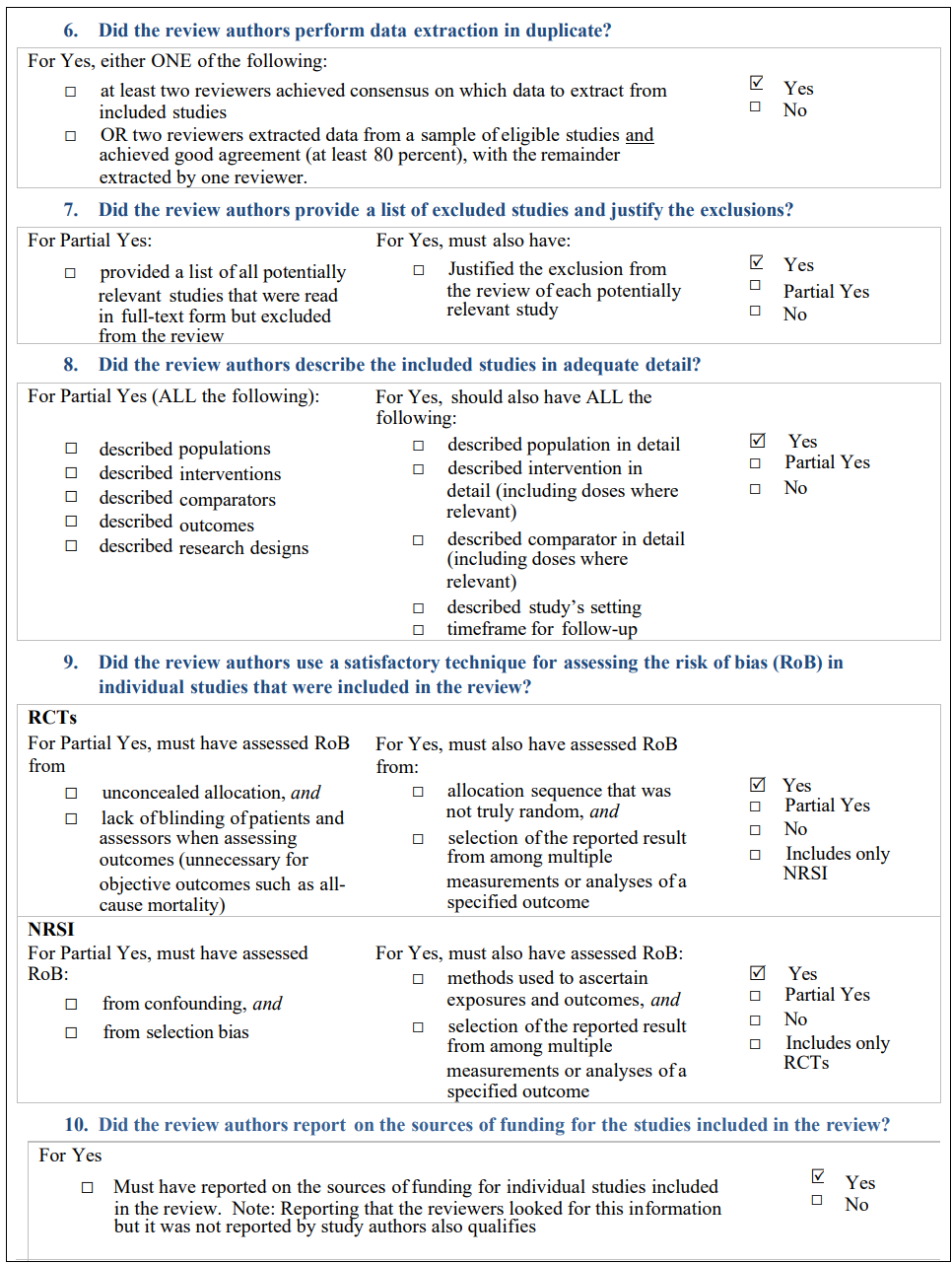

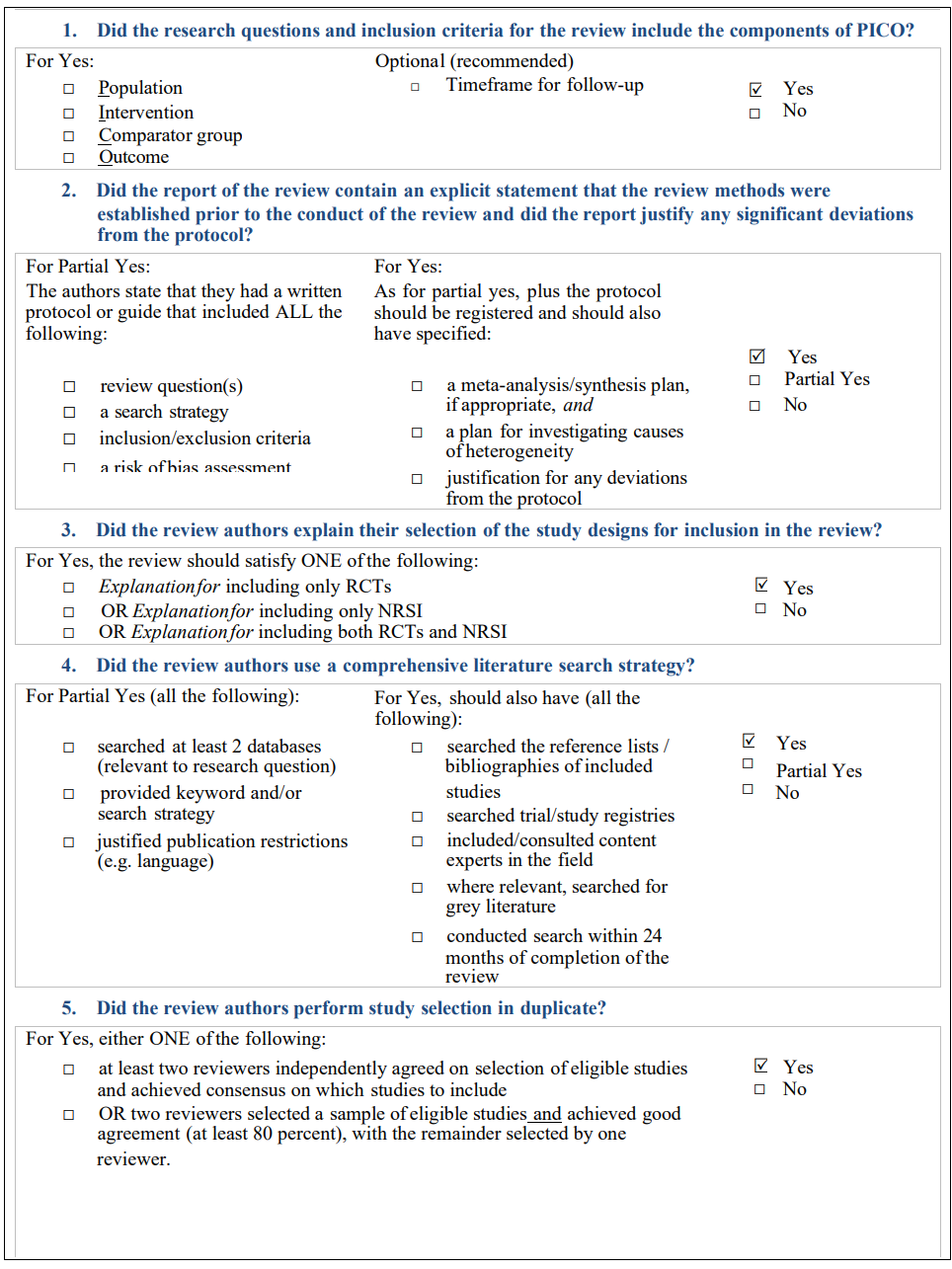
eFigure 1. AMSTAR Standard**

**
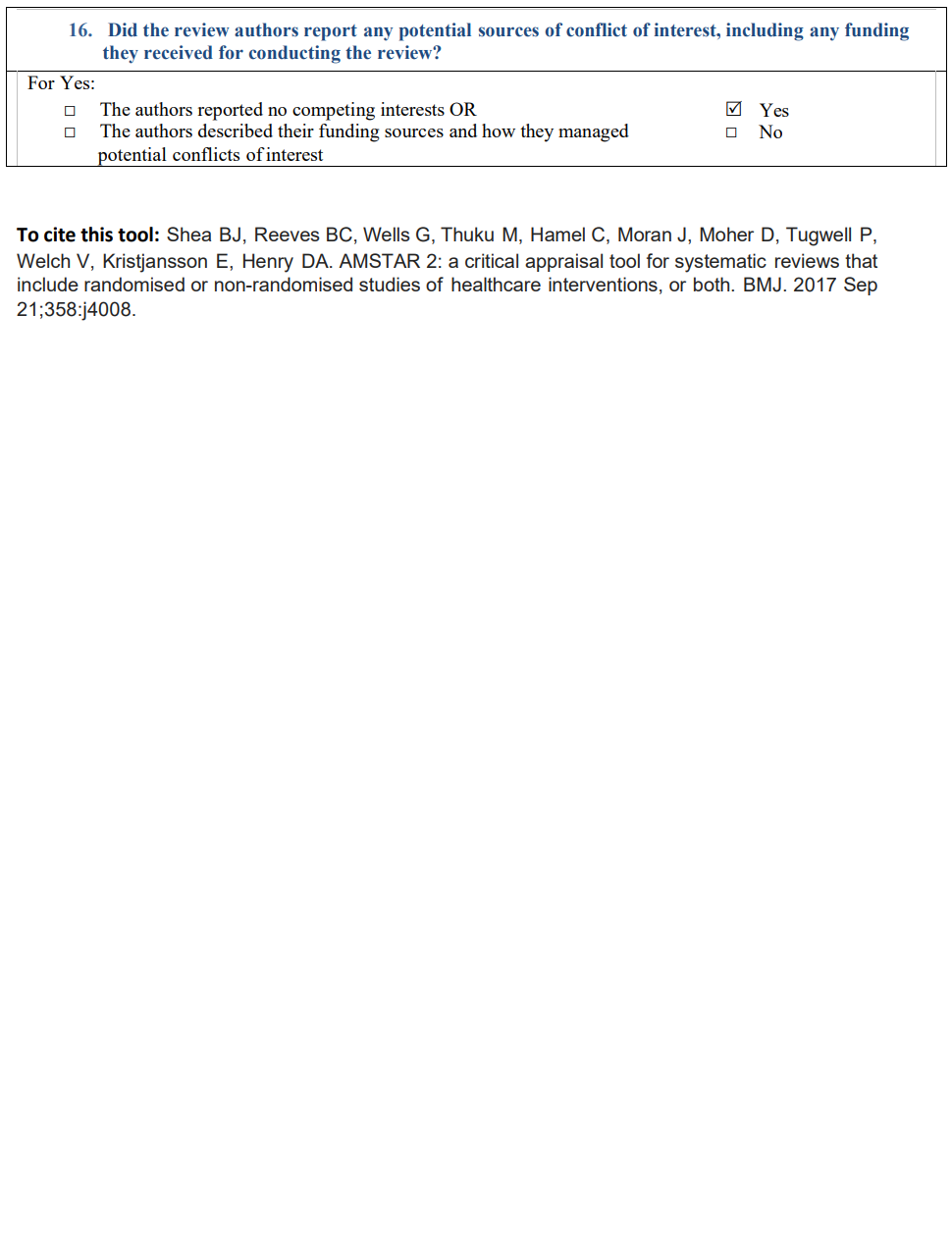

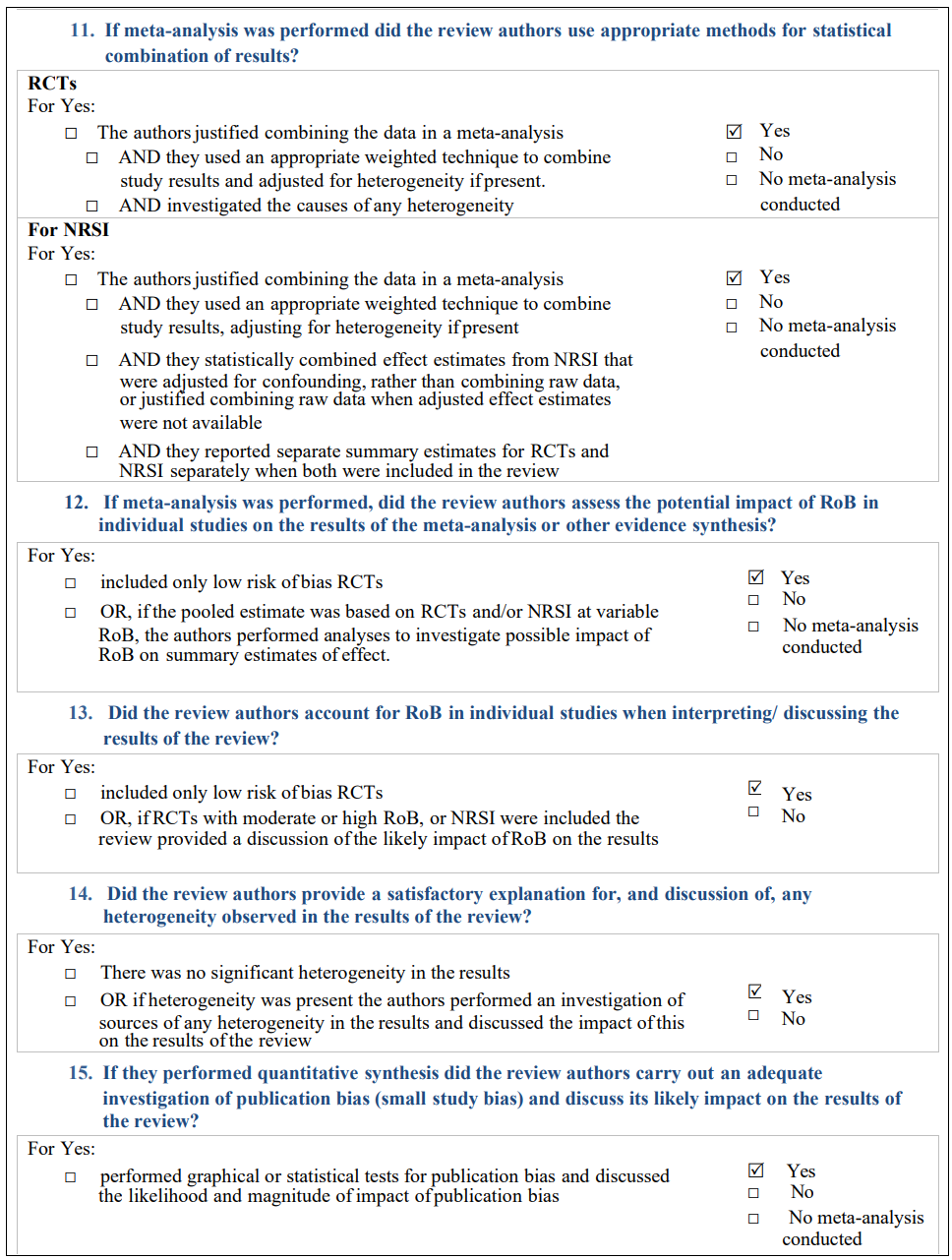
**

**eFigure 2. Forest plots of the incidence of NAEs**

-
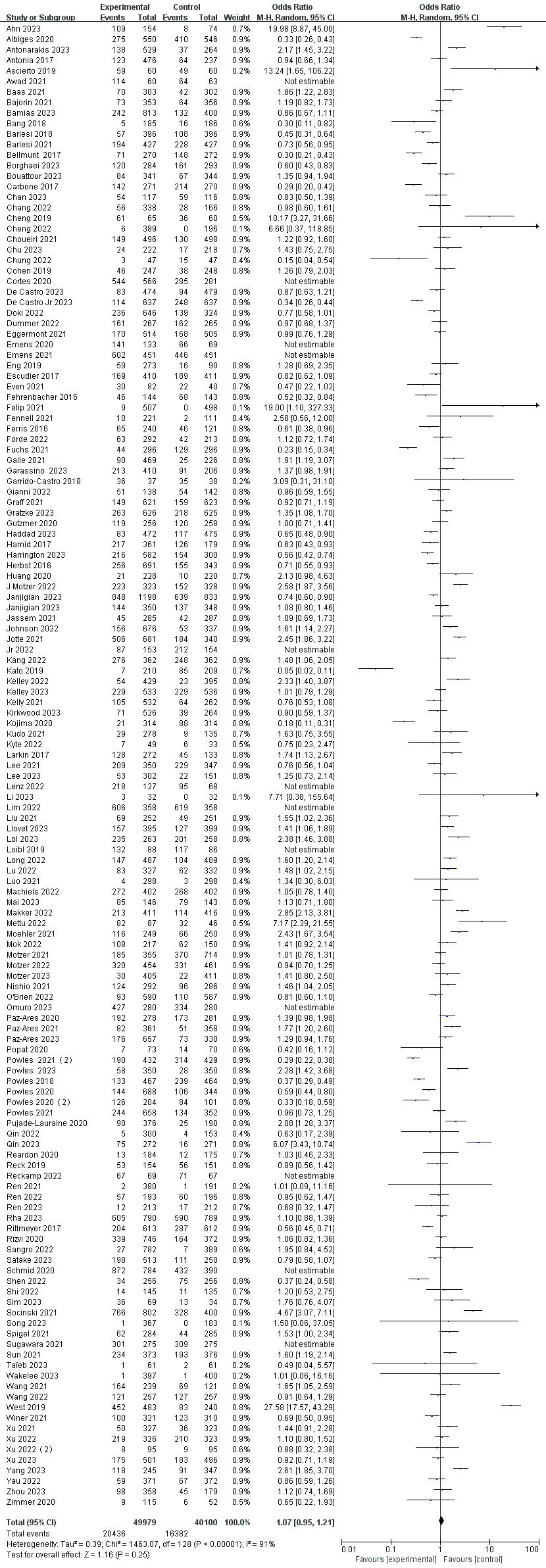

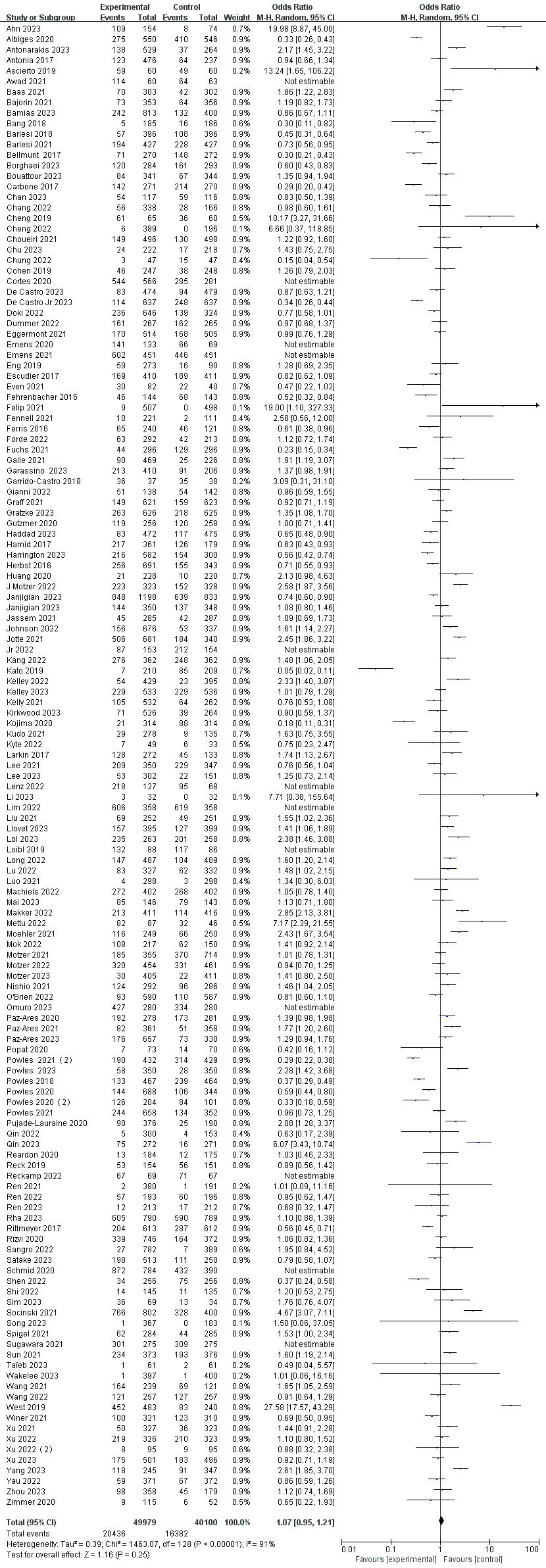
**Overall of toxicities**
-
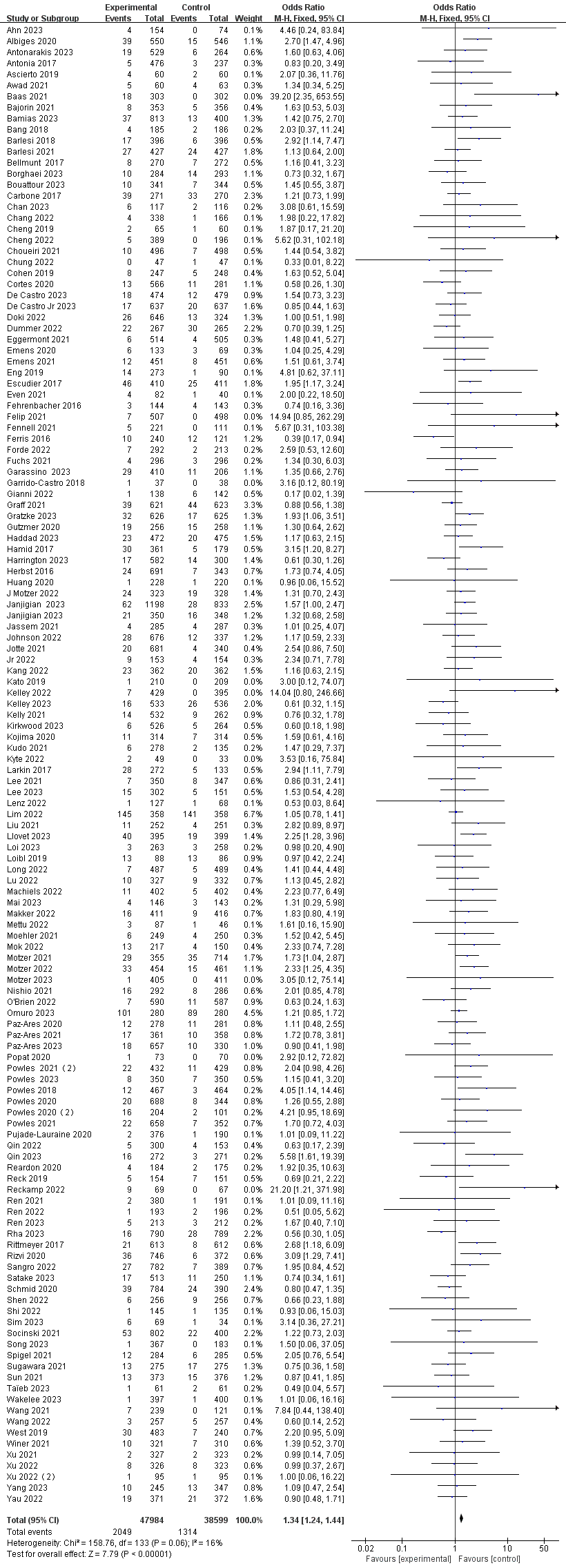

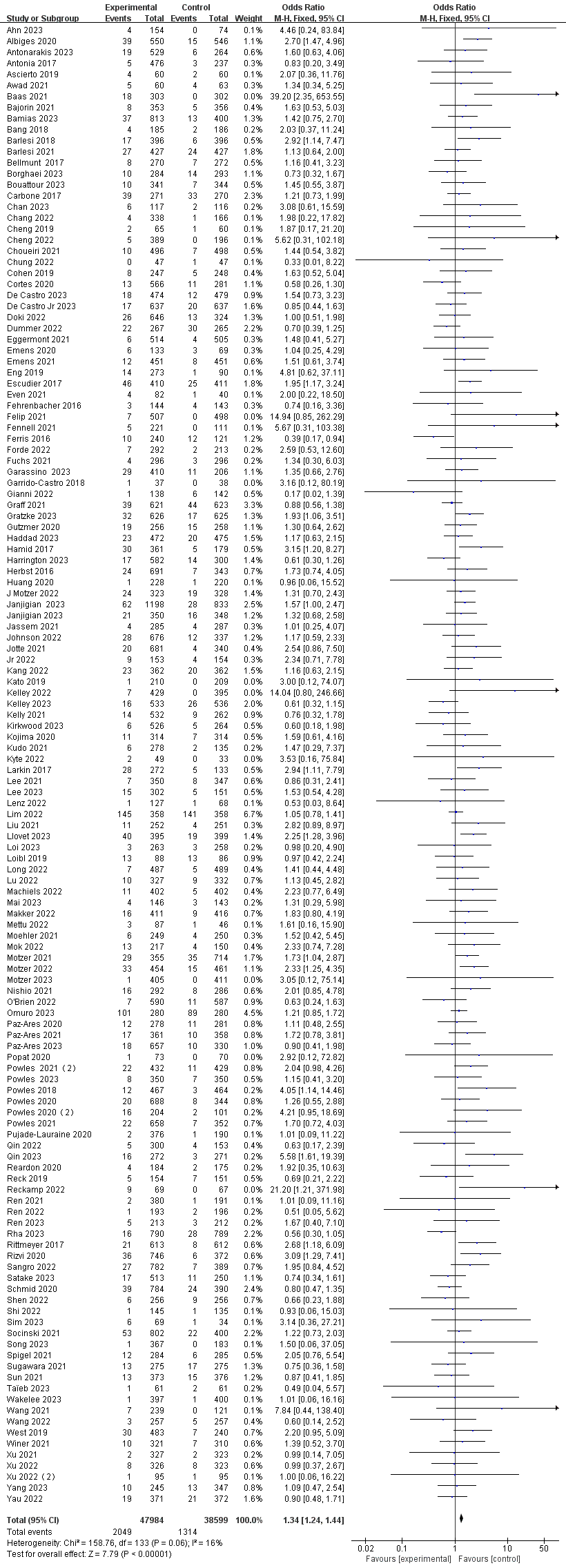
**Subgroup by severity of toxicities**
- **Subgroup by PD-1/PD-L1 inhibitor types**

**
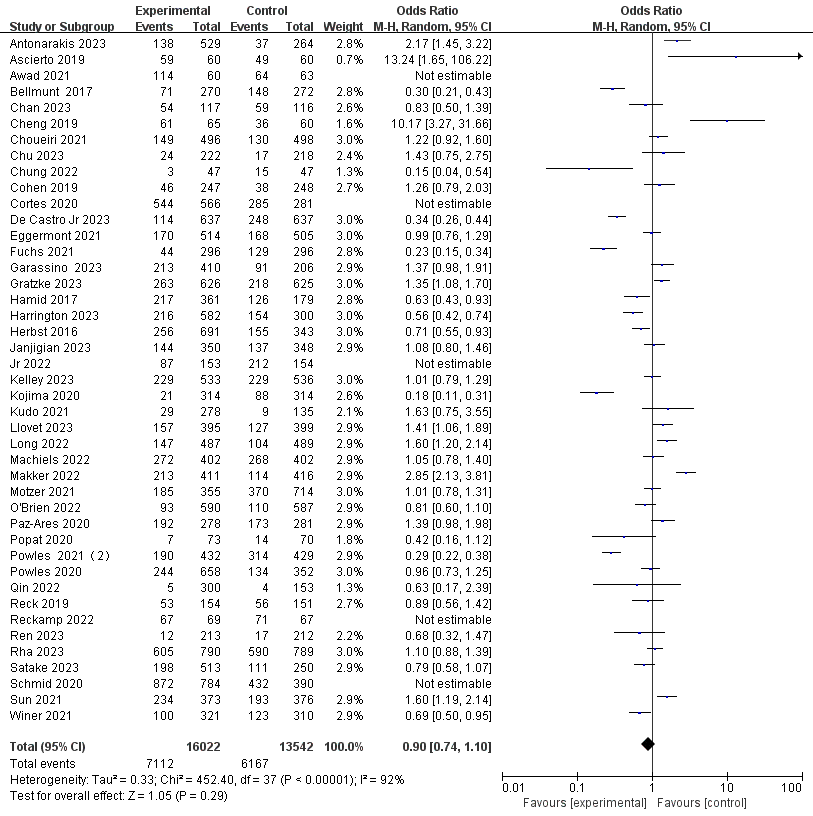
Pembrolizumab**

**Nivolumab**
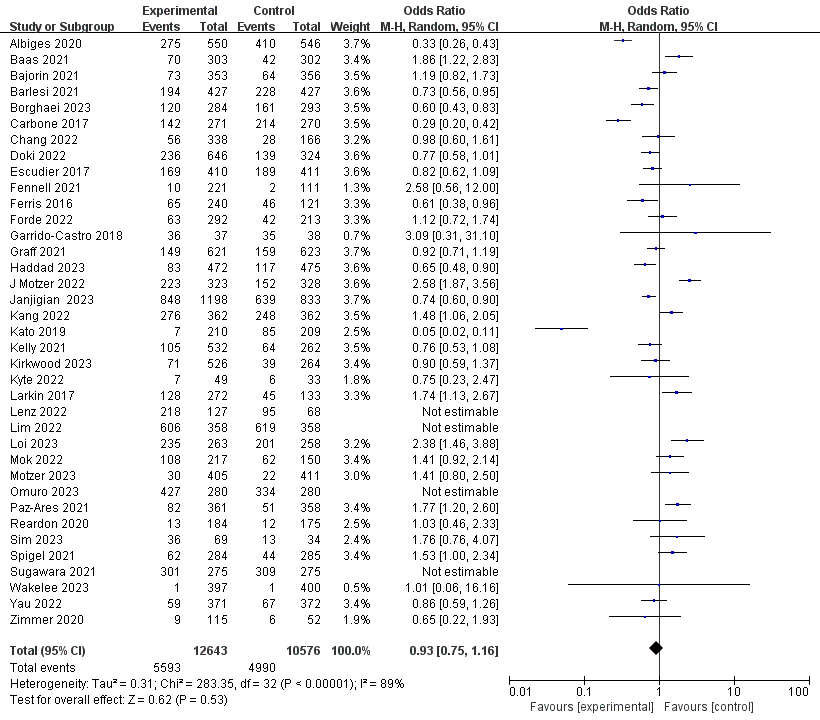


**
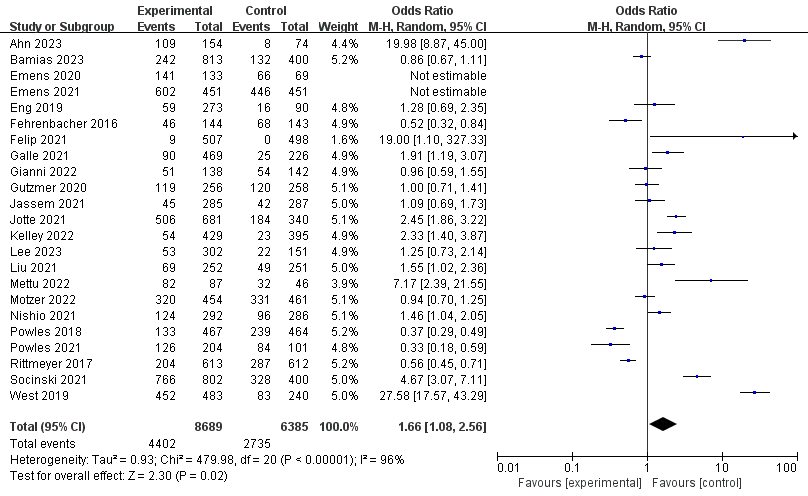
Atezolizumab**

- **Subgroup by cancer type**

**
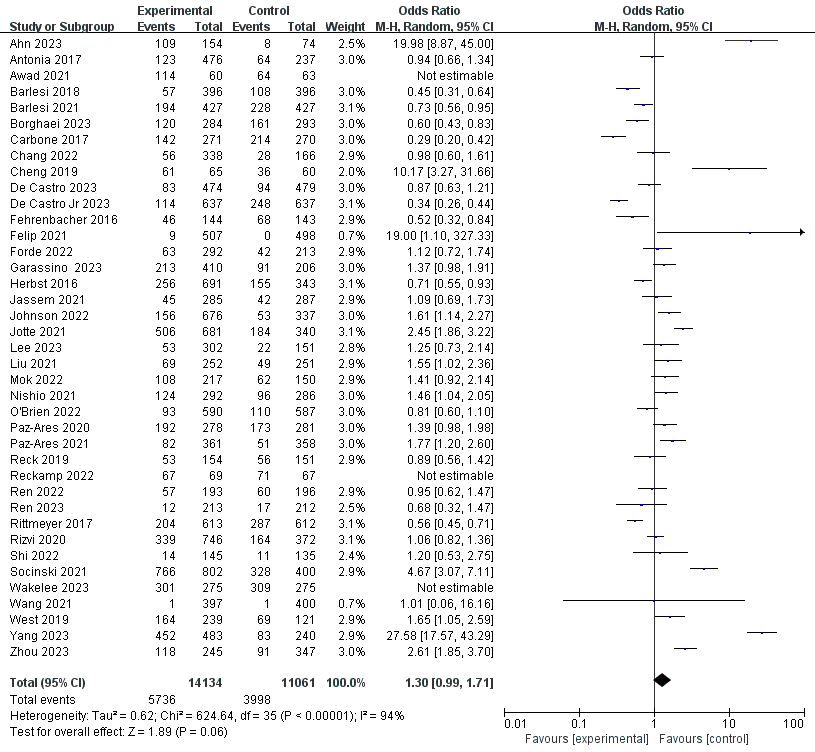
NSCLC**

**Esophageal Squamous Cell Carcinoma**


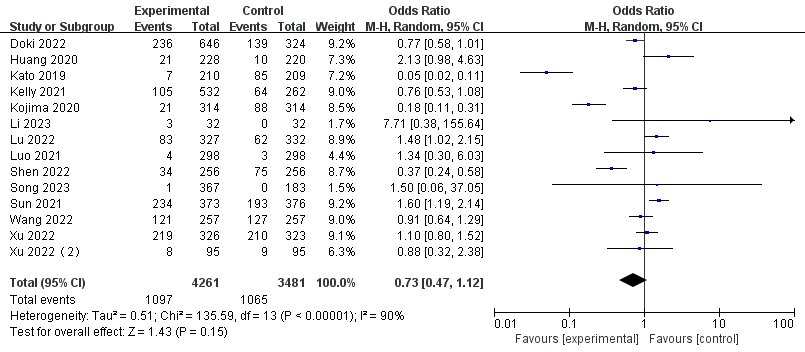


**Gastric Cancer**

**
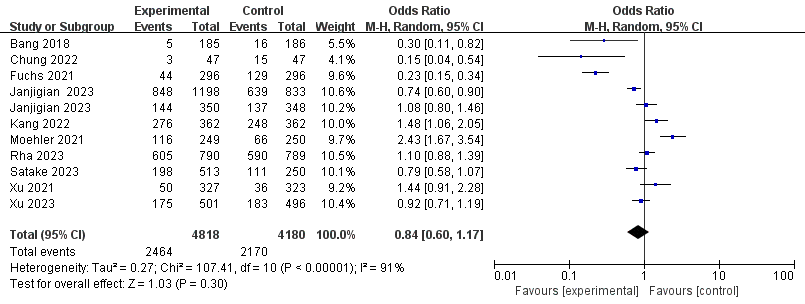
**

- **Subgroup by countries**

**
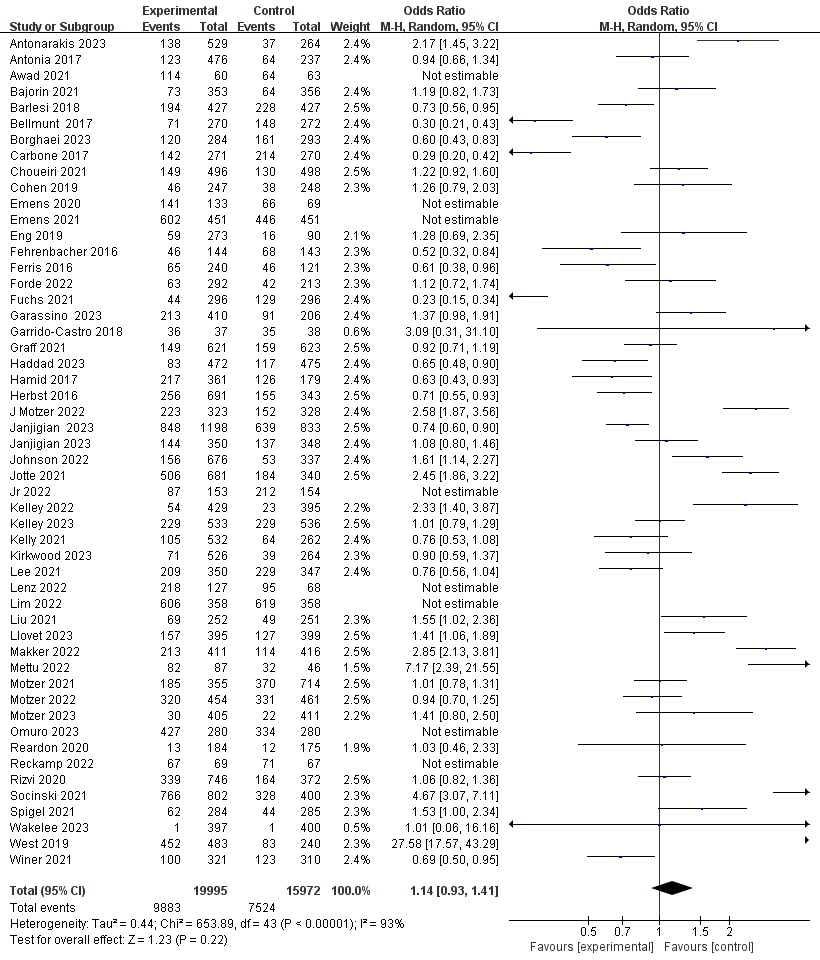
USA**

**China**

**
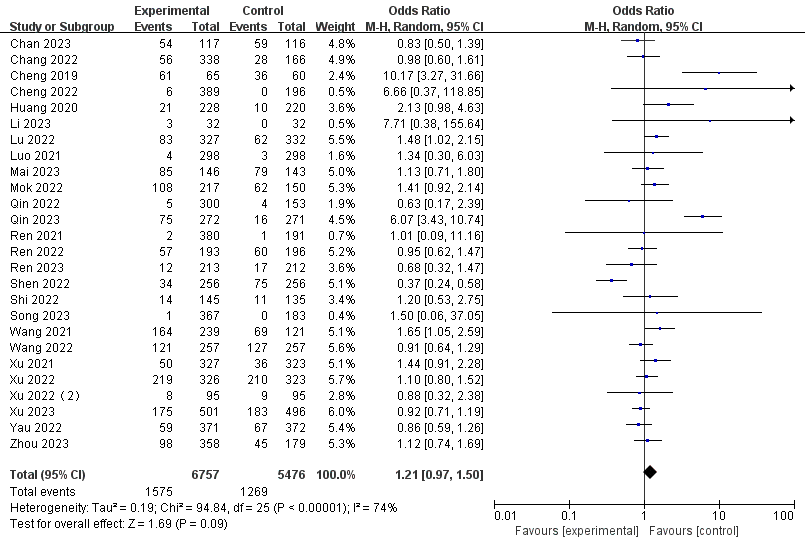
**

**UK**

**
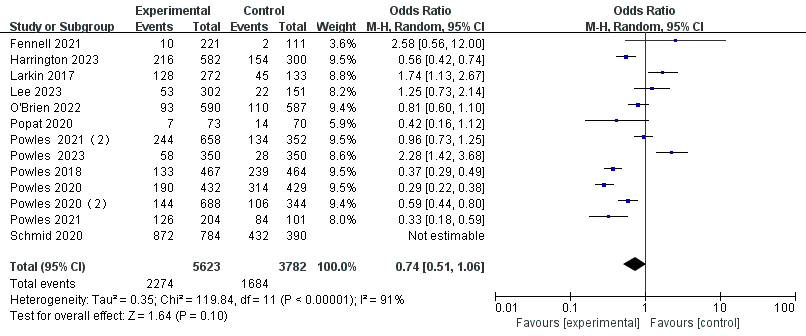
**

**eMethods. Search strategy**

- **PUBMED（23.11.27）——619**

**Search:** (("cancer" OR "tumor" OR "tumour" OR "neoplasm" OR "carcinoma") AND ("immune checkpoint inhibitor" OR "programmed death receptor 1" OR "programmed cell death 1" OR "anti-PD-1" OR "PD-1" OR "anti-PD-L1" OR "PD-L1" OR "nivolumab" OR "pembrolizumab" OR "atezolizumab" OR "durvalumab" OR "avelumab" OR "immunotherapy")) AND ("irAEs" OR "immune-related adverse events" OR "treatment-related adverse events" OR "treatment-related AEs" OR "select adverse events" OR "select AEs" OR "immune-mediated adverse events" OR "immune-mediated AEs")

**Filters:** Clinical Trial, Phase II, Clinical Trial, Phase III, Clinical Trial, Phase IV, Controlled Clinical Trial, Randomized Controlled Trial, Humans

(("cancer"[All Fields] OR "tumor"[All Fields] OR "tumour"[All Fields] OR "neoplasm"[All Fields] OR "carcinoma"[All Fields]) AND ("immune checkpoint inhibitor"[All Fields] OR "programmed death receptor 1"[All Fields] OR "programmed cell death 1"[All Fields] OR "anti-PD-1"[All Fields] OR "PD-1"[All Fields] OR "anti-PD-L1"[All Fields] OR "PD-L1"[All Fields] OR "nivolumab"[All Fields] OR "pembrolizumab"[All Fields] OR "atezolizumab"[All Fields] OR "durvalumab"[All Fields] OR "avelumab"[All Fields] OR "immunotherapy"[All Fields]) AND ("irAEs"[All Fields] OR "immune-related adverse events"[All Fields] OR "treatment-related adverse events"[All Fields] OR "treatment-related AEs"[All Fields] OR "select adverse events"[All Fields] OR "select AEs"[All Fields] OR "immune-mediated adverse events"[All Fields] OR "immune-mediated AEs"[All Fields])) AND ((clinicaltrialphaseii[Filter] OR clinicaltrialphaseiii[Filter] OR clinicaltrialphaseiv[Filter] OR controlledclinicaltrial[Filter] OR randomizedcontrolledtrial[Filter]) AND (humans[Filter]))

- **Web of Science（23.11.27）——601**

((((((((((((TS=(randomized controlled trial)) OR TS=(controlled clinical trial)) OR TS=(randomized))) OR TS=(Controlled Clinical Trial)) OR TS=(trail))) OR TS=(PHASE II)) OR TS=(PHASE III)) AND TS=("cancer" OR "tumor" OR "tumour" OR "neoplasm" OR "carcinoma")) AND TS=("immune checkpoint inhibitor" OR "programmed death receptor 1" OR "programmed cell death 1" OR "anti-PD-1" OR "PD-1" OR "anti-PD-L1" OR "PD-L1" OR "nivolumab" OR "pembrolizumab" OR "atezolizumab" OR "durvalumab" OR "avelumab" OR "immunotherapy")) AND TS=("irAEs" OR "immune-related adverse events" OR "treatment-related adverse events" OR "treatment-related AEs" OR "select adverse events" OR "select AEs" OR "immune-mediated adverse events" OR "immune-mediated AEs")) AND ((DT==("CLINICAL TRIAL")) NOT (SILOID==("PPRN")))

- **Embase（23.12.11）——965**

('immune checkpoint inhibitor'/exp OR 'immune checkpoint inhibitor' OR 'programmed death receptor 1'/exp OR 'programmed death receptor 1' OR 'programmed cell death 1'/exp OR 'programmed cell death 1' OR 'anti-pd-1' OR 'pd-1'/exp OR 'pd-1' OR 'anti-pd-l1' OR 'pd-l1' OR 'nivolumab'/exp OR 'nivolumab' OR 'pembrolizumab'/exp OR 'pembrolizumab' OR 'atezolizumab'/exp OR 'atezolizumab' OR 'durvalumab'/exp OR 'durvalumab' OR 'avelumab'/exp OR 'avelumab' OR 'immunotherapy'/exp OR 'immunotherapy') AND ('iraes' OR 'immune-related adverse events' OR 'treatment-related adverse events' OR 'treatment-related aes' OR 'select adverse events' OR 'select aes' OR 'immune-mediated adverse events' OR 'immune-mediated aes') AND ('cancer'/exp OR 'cancer' OR 'tumor'/exp OR 'tumor' OR 'tumour'/exp OR 'tumour' OR 'neoplasm'/exp OR 'neoplasm' OR 'carcinoma'/exp OR 'carcinoma') AND [randomized controlled trial]/lim

- **Cochrane Library（23.12.11）——1318**

("cancer" OR "tumor" OR "tumour" OR "neoplasm" OR "carcinoma"):ti,ab,kw AND ("immune checkpoint inhibitor" OR "programmed death receptor 1" OR "programmed cell death 1" OR "anti-PD-1" OR "PD-1" OR "anti-PD-L1" OR "PD-L1" OR "nivolumab" OR "pembrolizumab" OR "atezolizumab" OR "durvalumab" OR "avelumab" OR "immunotherapy"):ti,ab,kw AND ("irAEs" OR "immune-related adverse events" OR "treatment-related adverse events" OR "treatment-related AEs" OR "select adverse events" OR "select AEs" OR "immune-mediated adverse events" OR "immune-mediated AEs"):ti,ab,kw AND (“randomized controlled trial” OR “controlled clinical trial” OR “randomized” OR “Controlled Clinical Trial” OR “trail” OR “PHASE II” OR “PHASE III”):ti,ab,kw" in Trials (Word variations have been searched)
